# Overexpression of Two New Acyl-CoA:Diacylglycerol Acyltransferase 2-Like Acyl-CoA:Sterol Acyltransferases Enhanced Squalene Accumulation in *Aurantiochytrium limacinum*

**DOI:** 10.3389/fmicb.2022.822254

**Published:** 2022-01-25

**Authors:** E-Ming Rau, Zdenka Bartosova, Kåre Andre Kristiansen, Inga Marie Aasen, Per Bruheim, Helga Ertesvåg

**Affiliations:** ^1^Department of Biotechnology and Food Science, NTNU Norwegian University of Science and Technology, Trondheim, Norway; ^2^Department of Biotechnology and Nanomedicine, SINTEF Industry, Trondheim, Norway

**Keywords:** *Aurantiochytrium*, thraustochytrids, steryl esters, diacylglycerol acyltransferase, sterol acyltransferases, squalene, docosahexaenoic acid

## Abstract

Thraustochytrids are heterotrophic marine eukaryotes known to accumulate large amounts of triacylglycerols, and they also synthesize terpenoids like carotenoids and squalene, which all have an increasing market demand. However, a more extensive knowledge of the lipid metabolism is needed to develop thraustochytrids for profitable biomanufacturing. In this study, two putative type-2 Acyl-CoA:diacylglycerol acyltransferases (DGAT2) genes of *Aurantiochytrium* sp. T66, *T66ASATa*, and *T66ASATb*, and their homologs in *Aurantiochytrium limacinum* SR21, *AlASATa* and *AlASATb*, were characterized. In *A. limacinum* SR21, genomic knockout of *AlASATb* reduced the amount of the steryl esters of palmitic acid, SE (16:0), and docosahexaenoic acid, SE (22:6). The double mutant of *AlASATa* and *AlASATb* produced even less of these steryl esters. The expression and overexpression of *T66ASATb* and *AlASATb*, respectively, enhanced SE (16:0) and SE (22:6) production more significantly than those of *T66ASATa* and *AlASATa*. In contrast, these mutations did not significantly change the level of triacylglycerols or other lipid classes. The results suggest that the four genes encoded proteins possessing acyl-CoA:sterol acyltransferase (ASAT) activity synthesizing both SE (16:0) and SE (22:6), but with the contribution from AlASATb and T66ASATb being more important than that of AlASATa and T66ASATa. Furthermore, the expression and overexpression of *T66ASATb* and *AlASATb* enhanced squalene accumulation in SR21 by up to 88%. The discovery highlights the functional diversity of DGAT2-like proteins and provides valuable information on steryl ester and squalene synthesis in thraustochytrids, paving the way to enhance squalene production through metabolic engineering.

## Introduction

**FIGURE 1:**
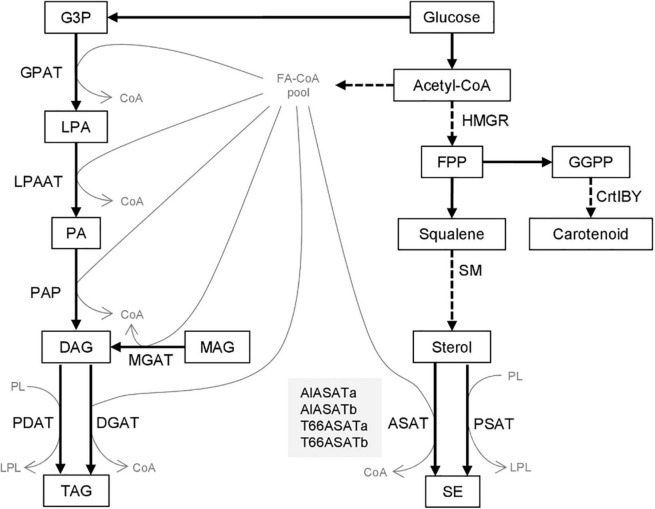
Biosynthesis of triacylglycerol and steryl esters from glucose. Compounds are shown as boxes or thin gray letters, enzymes by the arrows. Dashed arrows denote pathways where more than one enzymatic step are involved. The four proteins discussed in this paper are shown in the gray box. G3P, Glycerol-3-phosphate; LPA, lysophosphatidic acid; PA, phosphatidic acid; DAG, diacylglycerol; MAG, monoacylglycerol; TAG, triacylglycerol; PL, phospholipid; LPL, lysophospholipid; FA-CoA, Fatty-acyl-CoA; FPP, farnesyl pyrophosphate; GGPP, geranylgeranyl pyrophosphate; SE, steryl ester. GPAT, acyl-CoA:sn-glycerol-3-phosphate acyltransferase; LPAAT, acyl-CoA:lysophosphatidic acid acyltransferase; PAP, phosphatidic acid phosphatase; MGAT, acyl-CoA:monoacylglycerol acyltransferase; PDAT, phospholipid:diacylglycerol acyltransferase; DGAT, acyl-CoA:diacylglycerol acyltransferase; HMGR, β-Hydroxy β-methylglutaryl-CoA reductase; SM, squalene mono-oxygenase; ASAT, acyl-CoA:sterol acyltransferase; PSAT, phospholipid:sterol acyltransferase; CrtIBY, a trifunctional protein with phytoene desaturase; geranylgeranyl phytoene synthase and lycopene cyclase activities.

Fatty acids can be stored in lipid bodies as neutral triacylglycerol (TAG) or steryl ester (SE) and serve as energy or membrane material reservoirs (Liu et al., 2012; Korber et al., 2017). TAG can be synthesized by the sn-glycerol-3-phosphate (G3P) pathway starting from G3P as the backbone, followed by steps including three acylations with the acyl groups provided by fatty acyl-CoA (Figure 1). The final acylation of diacylglycerol (DAG) generates TAG, usually catalyzed by acyl-CoA:diacylglycerol acyltransferase (DGAT). In animals, it has been shown that DAG can also be produced from monoacylglycerol (MAG) by acyl-CoA:monoacylglycerol acyltransferase (MGAT) (Liu et al., 2012). The acyl-groups may also be donated by phosphatidylcholine, in reactions catalyzed by a phospholipid: diacylglycerol acyl transferase (PDAT). Similarly, SE can be synthesized by the acylation of the hydroxyl group in the C3-position of sterols with acyl-CoA as acyl donor, catalyzed by acyl-CoA:sterol acyltransferase (ASAT) or with an acyl group transferred from a phospholipid by phospholipid:sterol acyltransferase (PSAT) (Korber et al., 2017). Acetyl-CoA serves as a precursor for both fatty acids (FAs) and sterols (Figure 1).

Eukaryotic DGATs can be classified into five families. DGAT1 and DGAT2 are the two families that have been identified in most species, while DGAT3 and phytyl ester synthases (PES) families are more prevalent in plants, and the wax ester synthase/DGAT (WS/DGAT) family is more prevalent in bacteria. All DGATs but the soluble DGAT3s are integral membrane proteins (McFie et al., 2010; Liu et al., 2011; Hernández et al., 2012; Xu et al., 2021). The DGAT families do not share significant protein sequence similarities, but some are homologous to other acyltransferase families. DGAT1s belong to the membrane-bound O-acyl transferase (MBOAT) family, as do ASATs such as ACAT1 and ACAT2 in humans and ARE1 and ARE2 in yeast. On the other hand, DGAT2s are homologous to MGATs (Chang et al., 2011; Liu et al., 2012). Some microalgal species contain multiple *DGAT2* and *DGAT2*-like genes that show a high degree of diversity within the same species, suggesting that they evolved from multiple origins of the various DGAT2 isoforms (Chen and Smith, 2012).

Thraustochytrids are heterotrophic marine eukaryotic microbes that accumulate a large amount of docosahexaenoic acid (DHA, C22:6 n-3)-containing lipids, and also terpenoids like carotenoids and squalene, all of which have high commercial values (Aasen et al., 2016; Du et al., 2021). Squalene has been applied substantially in medical and food industries as an anticarcinogen, antioxidant and vaccine adjuvant (Gohil et al., 2019). Still, most proteins necessary for lipid and terpenoid biosynthesis in thraustochytrids have been annotated based on their similarity to characterized proteins in other model species, and the homology are often fairly low. Our interest in DGATs originated from the question, would different DGATs be responsible for attaching different fatty acyl CoAs? One DGAT2 and two WS/DGATs have been identified in *Thraustochytrium aureum* and *Thraustochytrium roseum*, respectively, all three were shown to produce both wax esters and TAGs by heterologous expression in *Saccharomyces cerevisiae* or *Arabidopsis thaliana* (Zhang et al., 2013; Zhang N. et al., 2017).

In the thraustochytrid *Aurantiochytrium* sp. T66 (hereafter called T66), two of the seven *DGAT-*like genes were found to have increased expression in the lipid accumulation phase (Heggeset et al., 2019), and this suggested that they could be involved in the biosynthesis of TAGs that are accumulated in this phase. Since T66 is not amenable to transformation, these two genes and their corresponding homologs (Table 1) in the *Aurantiochytrium limacinum* SR21 (hereafter called SR21) were investigated to identify their functions related to lipid accumulation. Based on the results of the reported study, these DGAT2-like proteins are most likely to function as ASATs, and were thus designated *T66ASATa*, *T66ASATb*, *AlASATa*, and *AlASATb*.

**TABLE 1:** Putative *DGAT-*like encoding genes in T66 (Heggeset et al., 2019), and their homologs in SR21 (JGI MycoCosm database).

*Aurantiochytrium* sp. T66	*Aurantiochytrium limacinum* SR21	Protein Identify (%)
T66002778.1 (*T66ASATa*)	Aurli1_137698 (*AlASATa*)	55
T66003952.1 (*T66ASATb*)	Aurli1_43553 (*AlASATb*)	68
T66011424.1	Aurli1_84317	81
T66007335.1	Aurli1_115416	80
T66010588.1	Aurli1_4822	72
T66004124.1	Aurli1_4822	44
T66001158.1	Aurli1_4822	44

*The identities of the encoded proteins were determined by NCBI BLASTp.*

## Materials and Methods

### Strains and Medium

*Aurantiochytrium limacinum* SR21 (ATCC MYA-1381) and *Aurantiochytrium* sp. T66 (ATCC PRA-276) and strains generated in this study (Supplementary Table 1) were cultivated and stored as described earlier (Rau et al., 2021).

### Phylogenetic Analysis

The analyses were conducted using MEGA X (Kumar et al., 2018) by using the Maximum Likelihood method and JTT matrix-based model (Jones et al., 1992). The analysis involved 50 proteins. Initial trees for the heuristic search were obtained automatically by applying Neighbor-Join and BioNJ algorithms to a matrix of pairwise distances estimated using the JTT model, and then selecting the topology with superior log likelihood value. The bootstrap consensus tree is taken to represent the evolutionary history of the taxa analyzed (Felsenstein, 1985).

### Plasmid Construction

Molecular cloning procedures and materials were performed as described earlier (Rau et al., 2021). NEBuilder^®^ HiFi DNA Assembly Master Mix (New England Biolabs, United States) was used for Gibson assembly, Plasmids, primers and gene accession numbers for the cloned genes are described in Supplementary Tables 2–4, respectively.

Supplementary Figure 1 provides a detailed description of how the plasmids for gene knock-outs were constructed. They contain antibiotic resistance cassettes flanked by two DNA fragments within the coding region or the upstream of the coding region in *AlASATa* or *AlASATb*. The antibiotic resistance cassettes contained either a Zeocin resistance gene (*ble*) or nourseothricin resistance gene (*nat*), in both cases controlled by the endogenous glyceraldehyde 3-phosphate dehydrogenase (GAPDH) promoter and terminator. pUC19_GZG (Faktorová et al., 2020) encoding the Zeocin resistance cassette was a gift from Jackie Collier, Stony Brook University.^1^ The fragment containing *nat* was amplified from pAG36 (Goldstein and McCusker, 1999), which was a gift from John McCusker,^2^ and transferred to pUC19_GZG by SLIC cloning (Islam et al., 2017).

The plasmids for ASAT expression, pEMR34, pEMR35, pEMR36, and pEMR37 contain *ble* linked to *AlASATa*, *AlASATb*, *T66ASATa*, or *T66ASATb* by a 2A peptide-encoding DNA fragment, respectively. A cleavage can occur at the 2A peptide during translation to produce two separated proteins. DNA fragment (r)GAPp-*ble*-2A-GOI-(f)GAPt was generated by overlap extension PCR (Hilgarth and Lanigan, 2020). Each expression cassette was controlled by the endogenous GAPDH promoter and terminator, flanked by two DNA fragments from the β-carotene synthesis gene *CrtIBY* (Aurli1_150841). Supplementary Figure 2 provides a detailed description of their construction. The disruption of *crtIBY* reduces the carotenoids synthesis without affecting cell viability and growth (Rius, 2021). Integration into *crtIBY* can thus be verified by the color of the colonies changing from brown to pale.

### Transformation of *Aurantiochytrium limacinum* SR21

The electrotransformation protocol of *A. limacinum* SR21 followed the procedures described earlier (Rau et al., 2021). Briefly, subcultured cells with OD_600_ ∼3 were collected and washed once by BSS (10 mM KCl, 10 mM NaCl, 3 mM CaCl_2_) and twice by 50 mM sucrose. The cells were then resuspended in 50 mM sucrose and transferred to 2-mm-gap cuvettes on ice. The volumes were adjusted to obtain an impedance (kΩ) between 0.9 and 1.5, measured by NEPA21 Electroporator (Nepa Gene Co., Ltd., Japan). Linearized DNA was added to the cell suspension in 2-mm-gap cuvettes, pulsed by the NEPA21 Electroporator with poring pulse parameters: two pulses, 275 V, 8 ms pulse length, 50 ms length interval, 10% decay rate, “+” polarity and transfer pulse parameters: one pulse, 20 V, 50 ms pulse length, 50 ms interval, 40% decay rate, “±” polarity. GPYS medium (3% glucose, 0.6% peptone, 0.2% yeast extract, 50 mM sucrose, 1.8% ocean salt) with antibiotics (200 μg/ml ampicillin, 200 μg/ml streptomycin), given as a preventative measure against bacterial contamination, was added to the cells immediately, and they were incubated overnight. The transformants were selected by plating on GPYS agar plates containing 100 μg/ml Zeocin (Thermo Fisher Scientific, United States) or nourseothricin (Jena Bioscience GmbH, Germany), and verified by PCR on extracted genomic DNA. WT SR21 was transformed using pEMR34, pEMR35, pEMR36, pEMR37, pEMR38, and pEMR39 to generate strains AlASATa-OE, AlASATb-OE, T66ASATa-KI, T66ASATb-KI, AlASATa-KO, and AlASATb-KO, respectively, that could grow on plates containing Zeocin. To generate a double mutant strain by disrupting *AlASATa* in strain AlASATb-KO, pEMR40 was used to transform AlASATb-KO cells generating strain AlASATab-KO that could grow on plates containing either nourseothricin or Zeocin.

### RT-PCR Analysis

RNA isolation, cDNAs synthesis and RT-PCR were performed as described earlier (Rau et al., 2021) using the β-tubulin gene cDNA as a reference gene (Zhang K. et al., 2017). PCR was performed to amplify parts of *AlASATa* cDNA by the primer pair 137698RT_f and 137698RT_r, *AlASATb* cDNA by the primer pair 43553RT_f and 43553RT_r, and β-tubulin gene cDNA by the primer pair SR21tubF2 and SR21tubR2 (Supplementary Table 3). To rule out genomic DNA contamination of the isolated RNA, products from cDNAs synthesis reactions with no reverse transcriptase were included as PCR templates.

### Cultivations for Lipid Profiling

Single colonies were inoculated in tubes with GPY medium at 28°C for 2 days with rotary shaking at 170 rpm, followed by sub-culturing cells with starting OD_600_∼0.01 using 100 ml lipid accumulation medium (Rau et al., 2021) in 500 ml baffled flasks and 170 rpm rotary shaking at 28°C. For FA analysis, 1.5 ml of the culture was stored at –20°C for each collection time point. For lipid extraction and determination of the lipid classes and species, 1.5 ml of the culture was centrifuged at 4,500 g for 1 min and washed with 3 ml mineral buffer (2 g/l NH_4_Cl, 18 g/l Na_2_SO_4_, 0.25 g/l MgSO_4_ × 7H_2_O, 0.4 g/l KCl, 6.1 g/l Tris-base and 5.8 g/l maleic acid) and then with 3 ml milli-Q water. The pellets were stored at –20°C.

### Lipid Extraction and Total Lipid Content

Lipid were extracted as described earlier (Bartosova et al., 2021a) with modifications. The cell pellets were snap-freezed by submerging them in liquid nitrogen for about 10 s and lyophilized overnight. Five milligram(s) of dried cell pellets were mixed with zirconium oxide beads (0.5 ± 0.01 g, Ø 1.4 mm) in 2 ml vials with 1 ml of a cold mixture of chloroform:methanol (1:2, v/v). The mixtures were homogenized with three bead-beating cycles at 6,500 rpm for 30 s with 15 s intermediate pause by a Precellys^®^ 24 bead homogenizer with a Cryolys temperature controller (all Bertin Technologies SAS, Montigny-le-Bretonneux, France). Cold chloroform 333 μl were then added, followed by vortexing for 20 s. Phase separation was induced by adding 333 μl of water, followed by vortexing for 20 s. The phase separation was accelerated by centrifuging at 14,000 rpm for 5 min at 15°C. A lower chloroform layer containing lipids was collected and cleared of cell debris with a syringe filter with PTFE membrane, 0.2 um, Ø 13 mm (VWR, United States). For determination of total lipids, 300 μl of the lipid extracts were transferred to a pre-weighed glass vial, left in fume hood for evaporation and weighed after 2 days.

The remaining extracts were flushed with a stream of nitrogen and stored at −80°C in dark glass vials for analyses of lipid classes and species.

### Lipid Species

The FA concentrations were determined by LC-MS (Heggeset et al., 2019). For determining lipid classes and species, lipid extracts were analyzed by a non-target semiquantitative lipidomics method based on ultrahigh performance supercritical fluid chromatography (UHPSFC)-mass spectrometry (MS) (Bartosova et al., 2021a,b). Dichloromethane was used as a sample diluent. The concentration of individual lipid classes and species in the lipid extracts was quantified using a single point standardization with one internal standard (ISTD) representative of known concentration for each lipid class. Strains were grouped as follows: KO (WT, AlASATa-KO, AlASATb-KO and AlASATab-KO), OE (30-1, AlASATa-OE and AlASATb-OE), and KI (30-1, T66ASATa-KI and T66ASATb-KI). The production (mg/g_*CDW*_) of a lipid specie was considered statistically significant different between the control strain (WT or 30-1) and one of the experimental strains (strains other than WT and 30-1) if *p* < 0.05, according to both one-way analysis of variance (ANOVA) for the group, followed by *post hoc* Tukey test, performed by MetaboAnalyst 5.0 (Pang et al., 2021), and unpaired *t*-test by using GraphPad.

The squalene concentrations of the samples were determined by UHPLC-MS/MS with squalene (S3626, Sigma-Aldrich, United States) as external standard. Fifty μl of lipid extract was vaporized under a stream of nitrogen at room temperature for about 10 min and then re-dissolved in 50 μL acetone. The samples were separated by UPLC as described earlier (Hakvåg et al., 2020) using a ACQUITY UPLC^®^ CSH C18 Column (pore size: 130 Å, length: 150 mm, inner diameter: 2.1 mm, particle size: 1.7 μm) (Waters, United States). Mobile phase A consisted of acetonitrile:methanol:isopropanol (80:15:5) and mobile phase B of isopropanol:acetonitrile (90:10), both containing 0.10 (v/v)% formic acid. Ethanol was used as needle wash, and the injection volume was 2 μl. MS/MS analyses were performed with a TQ-S mass spectrometer (Waters, United States) under APCI conditions. Corona pin current and source offset voltages were set to 5.0 μA and 50 V, respectively. Source temperature was maintained at 150°C, probe temperature 550°C and gas flow rate at 500 l/h. Cone gas flow rate was fixed at 150 l/h, and the nebulizer gas flow was maintained at 4.0 bar. Collision gas flow was set to 0.15 ml/min of argon. Cone voltages (CV), collision energies (CE) and MS/MS transitions (precursor and daughter ions) of squalene were optimized by infusing 5 μM squalene in acetone at 10–20 μl/min, combined with 0.05 ml/min with equal amounts of mobile phase A and B from the UHPLC-system. Squalene was quantified by means of one selected precursor ion-product ion transition (m/z 411.56–149.23, CV = 34 V, CE = 20 eV), and its identity confirmed by one transition (m/z 411.56–109.16, CV = 34 V, and CE = 26 eV). A 23 ms dwell time was calculated for each transition to ensure 25 data points across the peaks. UHPLC–MS/MS data were acquired and processed using MassLynx software (v4.1) and TargetLynx application manager.

## Results

### Phylogenetic Analysis of Diacylglycerol Acyltransferase -Like Proteins in SR21 and T66

**FIGURE 2:**
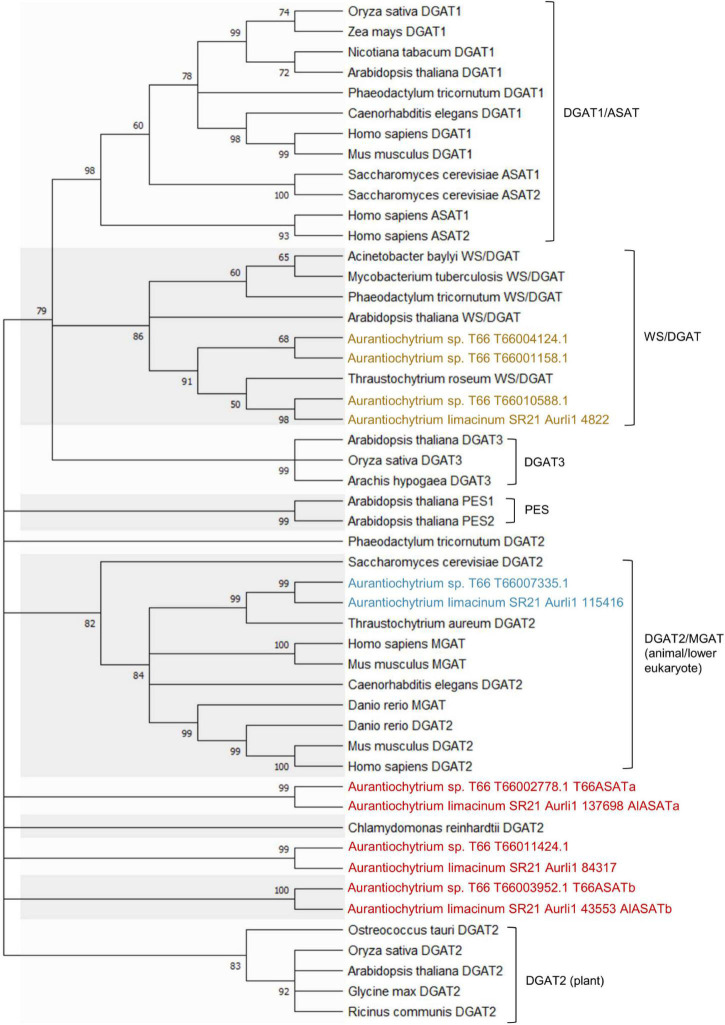
Phylogenetic analysis of DGAT2s (blue), WS/DGATs (yellow), and DGAT2-like proteins (red) in T66 and SR21, compared to selected DGAT-like proteins from different organisms. Supplementary Table 5 provides the Genbank accession number for each protein. The percentage of replicate trees in which the associated taxa clustered together in the bootstrap test (1,000 replicates) are shown next to the branches, which were collapsed if the corresponding partitions reproduced in less than 50% bootstrap replicates.

Seven genes were annotated to encode DGAT-like proteins in the T66 genome (Heggeset et al., 2019). A phylogenetic analysis was conducted, including the five putative DGAT-like homologs in SR21 (JGI MycoCosm database) (Table 1) and representatives from the different groups of DGATs from other organisms (Figure 2). The T66007335.1 and Aurli1_115416-encoded proteins were closely related to the functionally characterized TaDGAT2 from thraustochytrid *T. aureum* with protein identity 62 and 71%, respectively (Zhang et al., 2013), in a subcluster with DGAT2s from animals and *S. cerevisiae*. Similarly, the proteins encoded by T66010588.1, T66004124.1, T66001158.1 and Aurli1_4822 are clustered with TrWSD4 from *T. aureum* (Zhang N. et al., 2017) together with other known bifunctional WS/DGATs. On the other hand, AlASATa, T66ASATa, AlASATb, T66ASATb, and the *T66011424.1* and the *Aurli1_84317*-encoded proteins were found as relatively individual pairs within the large cluster of DGAT2 and PES, which we choose to call DGAT2-like proteins.

### Sequence Studies of the Selected Diacylglycerol Acyltransferase-Like Genes

**FIGURE 3:**
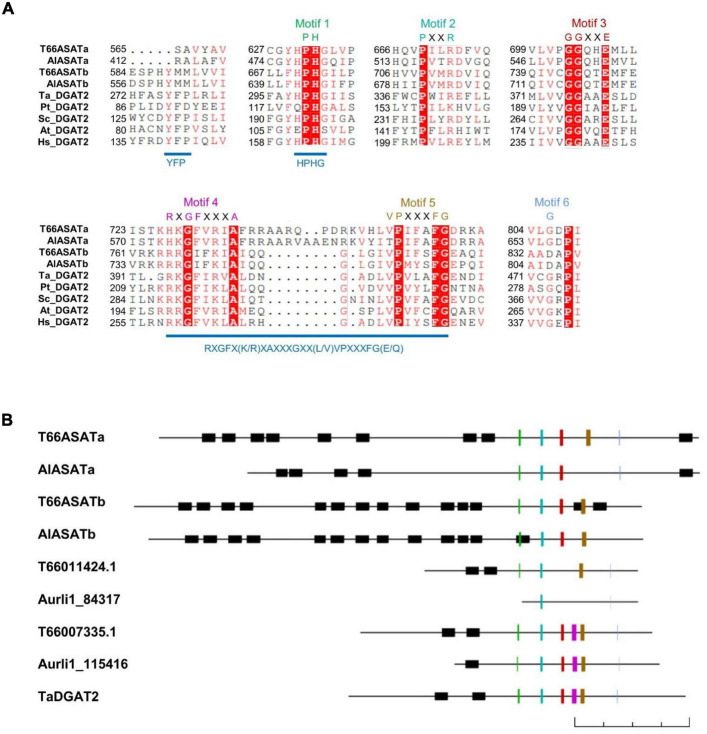
**(A)** Sequence alignment of T66ASATa, T66ASATb, AlASATa, and AlASATb with the conserved parts of DGAT2 from some species. The alignment was generated by ClustalW, illustrated by ESPript 3.0 (Robert and Gouet, 2014). The first two letters denote the species encoding the selected proteins from Figure 2: *T. aureum*, *P. tricornutum*, *S. cerevisiae*, *A. thaliana*, and *H. sapiens*. White letters with a red background denotes consensus residues, red letters: identical or similar residues, Motif 1–6: DGAT2 conserved motifs, see the text. **(B)** Schematic representation of the DGAT2-like proteins identified in T66 and SR21, and a function-verified TaDGAT2, oriented with the N-terminus to the left and the C-terminus to the right. All proteins are drawn in scale by Clone Manager with the lengths indicated by a 200 amino acids scale bar at the bottom. Black boxes: transmembrane domains predicted by TMHMM (Sonnhammer et al., 1998), colored lines: DGAT2 conserved Motif 1–6 with colors as indicated in **(A)**.

DGAT2 proteins are characterized by six conserved motifs (Cao, 2011). When the protein sequences of the four selected proteins were aligned with DGAT2s from other species (Figure 3A), all four were found to contain the DGAT2-conserved Motif 1 (PH block), Motif 2 (PR block) and Motif 3 (GGE block), while Motif 4 (RGFA block), Motif 5 (VPFG block) and Motif 6 (G block) were not all completely present in all four *Aurantiochytrium* enzymes. All four have the HPHG motif that was shown to be essential to murine DGAT2 enzyme activity (Stone et al., 2006; Liu et al., 2011), but not the YFP motif that is important to yeast DGAT2 activity (Liu et al., 2011). Another motif proposed to be important to DGAT2 catalysis, RXGFX(K/R)XAXXXGXX(L/V)VPXXXFG(E/Q) (Liu et al., 2012), is only partially conserved in the four enzymes.

AlASATa and T66ASATa share little sequence homology to AlASATb and T66ASATb, with protein identities of 28 and 25%, respectively. All four have the conserved regions mainly located at the C-terminal region, while their N-terminal parts are predicted to contain multiple transmembrane domains (TMDs), a structural feature that is more common in DGAT1s or ASATs, but rarely found in DGAT2s (Liu et al., 2012). All four were longer than other putative DGAT2s identified in T66 and SR21 (Figure 3B). If the residues from the N-terminal to the residue located 50 amino acids before the Motif 1 are removed, all four proteins appear to be clustered in the phylogenetic tree (Supplementary Figure 3). To date, no structure of any DGAT2s has been determined. The protein structures of AlASATa and AlASATb were subjected to homology modeling using Phyre2 (Kelley et al., 2015; Supplementary Figure 4), showing that the C-terminal part of the proteins shares structural conservation at > 99% confidence with PlsC, a bacterial GPAT (Robertson et al., 2017) although AlASATa and AlASATb have low sequence identities to PlsC with only 12 and 15%, respectively. On the other hand, the N-terminal regions of the four enzymes share no structural conservation with proteins in the PDB database. Taken together, all four proteins have sequence features absent in characterized DGAT2s, although all display several conserved DGAT2-specific motifs.

### The Effect of *AlASATa* and/or *AlASATb* Knockout on the Lipid Profile of the Mutants

To investigate the function of *AlASATa* and *AlASATb*, we chose to disrupt *AlASATa*, *AlASATb* in the SR21 genome with antibiotic resistance cassettes using homologous recombination, generating the single knock-out strains AlASATa-KO, AlASATb-KO and the double knock-out strain AlASATab-KO. Genomic DNA from strain AlASATa-KO, AlASATb-KO and AlASATab-KO were tested by PCR to confirm that the expected parts of *AlASATa* (386–543 bp from 5′), *AlASATb* (1,223–1,405 bp from 5′) and both genes, respectively, had been replaced by the antibiotic resistance cassette in a single copy (Supplementary Figures 5A,B, 6), which validated that the corresponding genes had been inactivated. No transcripts for the inactivated genes(s) were detected, providing further evidence of there being no wild-type gene copies left (Supplementary Figure 5C).

**FIGURE 4:**
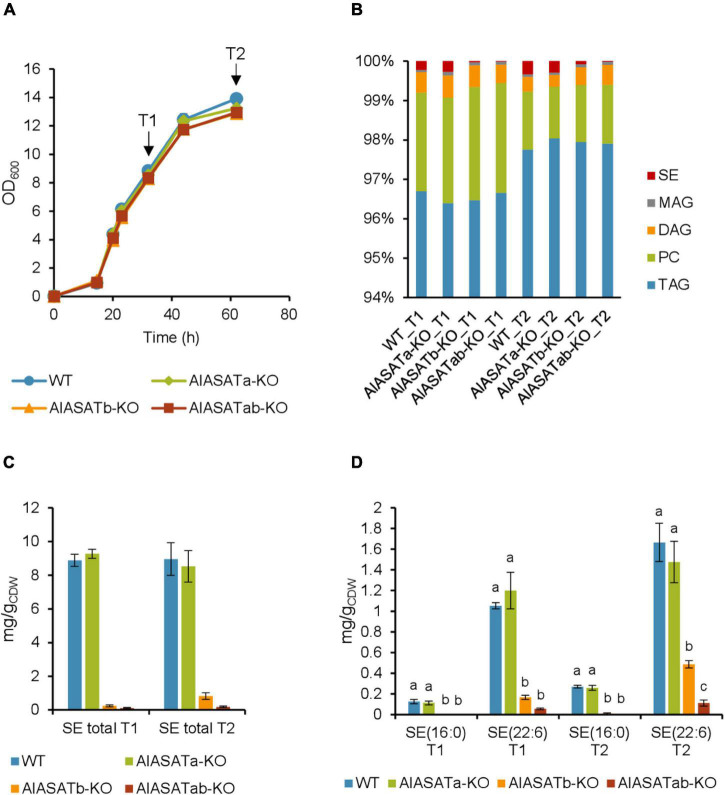
Characterization of the SR21 KO strains AlASATa-KO, AlASATb-KO and AlASATab-KO compared to WT. **(A)** Growth measured as optical density (OD_600_), sample collection points are indicated by arrows. **(B)** Distribution of different lipid classes. TAG, triacylglycerol; DAG, diacylglycerol; PC, phosphatidylcholine; MAG, monoacylglycerol; SE, steryl ester. The production (mg/g_*CDW*_) of total SE **(C)** and SE species **(D)** from the strains. All data are expressed as the mean of the triplicates originating from three independent cultures; Error bars represent the standard deviation. The bars are separated into groups, each with a particular SE and time points. For each group, the bars with different lowercase letters on the top are statistically significantly different from each other (*P* < 0.05), based on one-way analysis of variance (ANOVA), followed by *post hoc* Tukey test.

Strains SR21 WT, AlASATa-KO, AlASATb-KO, and AlASATab-KO displayed similar growth rates when they were cultivated in shake flasks using glucose as the carbon source (Figure 4A). Since it is known that thraustochytrid cells accumulate most of the lipids during nitrogen starvation (Heggeset et al., 2019), cells from early (T1) and late (T2) lipid accumulation phases were sampled (Figure 4A).

Analysis of the total FAs showed that the composition was not significantly different between the strains (Supplementary Table 6). Palmitic acid (C16:0) was the most abundant FA (∼58%), followed by DHA (C22:6 n-3) (∼27%). Deletion of DGATs could potentially decrease the amount of synthesized lipids, but the total lipid content was not significantly different between the selected strains (Supplementary Table 7). When the amount of the different lipid classes was analyzed, more than 96% of total lipid was triacylglycerol (TAG) (Figure 4B). The amounts of TAG, phosphatidylcholine (PC), and monoacylglycerol (MAG) were not significantly different between the different strains, but there was slightly more diacylglycerol (DAG) at T2 for the AlASATb-KO and AlASATab-KO (Supplementary Table 8). However, the SE levels were significantly lower in AlASATb-KO and AlASATab-KO, compared to WT. The SE level in AlASATab-KO was only 1∼2% of the level in WT (Figure 4C).

Since deletion of DGATs potentially can reduce the synthesis of particular lipid species, we further looked for the lipid species that showed statistically significant reduction in at least one of the mutant strains compared to WT at T1 or T2. No TAG species were significantly reduced, ruling out the possibility that these proteins are necessary to synthesize any minor TAG species. The species that showed significant reduction include the SEs of C16:0, SE (16:0) and the SEs of C22:6 n-3, SE (22:6) (Figure 4D and Supplementary Table 9). A third SE species with approximately the same abundance as SE (16:0) for all tested strains was also detected, but could not be assigned to any combination of FAs and sterols known to be synthesized by SR21 and is not discussed further. On the other hand, no lipid species showed statistically significant increases in any of the mutant strains compared to WT at T1 or T2. In AlASATb-KO, SE (16:0) could only be detected at T2. The production of SE (22:6) was less than 84 and 71% of that of the wild type for T1 and T2, respectively. Inactivation of AlASATa alone did not result in a significant decrease in any of the SE species. However, the amount of the SE (22:6) in AlASATab-KO was further decreased relatively to the AlASATb-KO strain (Figure 4D and Supplementary Table 9). These results suggest that both proteins have ASAT activity and that AlASATb is the main enzyme necessary for accumulation of the two SEs, while AlASATa is contributing to SE (22:6) accumulation when *AlASATb* is inactivated.

### The Effect of Overexpression of *AlASATa* or *AlASATb*, and Expression of *T66ASATa* or *T66ASATb* on the Level of Steryl Esters

While the results from the knockout strains clearly indicate a function for AlASATa and AlASATb, we wanted to verify this by expressing an additional copy of these genes in SR21. Moreover, we wanted to see if the T66 homologs, *T66ASATa* and *T66ASATb*, would have similar functions when expressed in SR21. Expression cassettes of *AlASATa*, *AlASATb*, *T66ASATa* or *T66ASATb* were integrated into the β-carotene synthesis gene *CrtIBY* in SR21 and generated the strain AlASATa-OE, AlASATb-OE, T66ASATa-KI or T66ASATb-KI, respectively. Genomic DNA from these strains was analyzed by PCR to demonstrate that parts of *crtIBY* (1,845–2,204 bp from 5′) had been replaced with the corresponding expression cassettes in a single copy (Supplementary Figures 7, 8). These strains also showed the expected pale phenotype (not shown), which further supported that the expression cassette was correctly integrated. Since all genes were to be expressed by the same GAPDH promoter and terminator (Supplementary Figure 7A) and inserted into the same genome location, it seemed most likely that any observed phenotypes would be due to the enzymatic or regulatory properties of the individual proteins. However, differences in transcript or protein stability cannot be ruled out.

**FIGURE 5:**
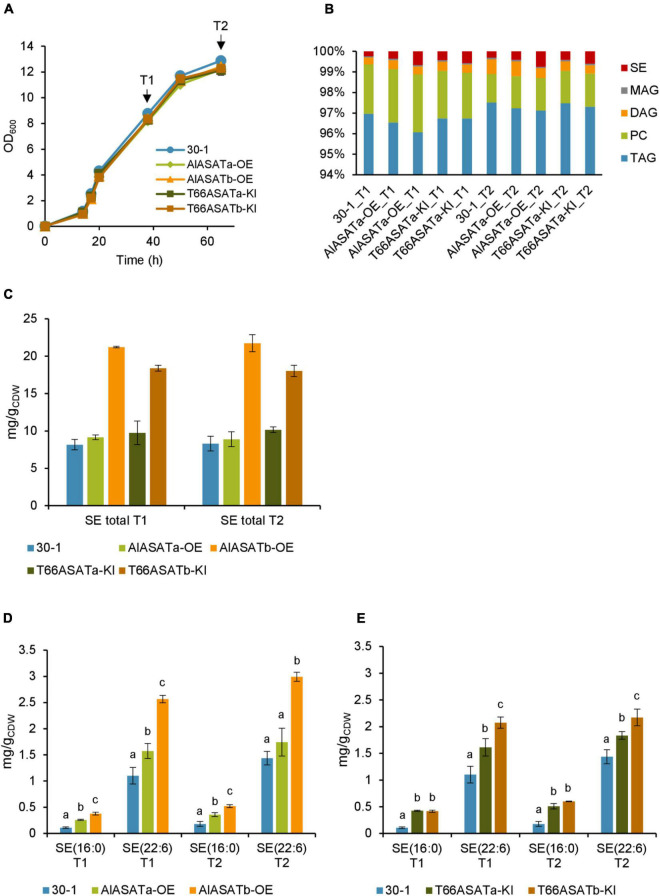
Characterization of the SR21 OE and KI strains compared to strain 30-1. **(A)** Growth measured as optical density (OD_600_), sample collection points are indicated by arrows. **(B)** Distribution of different lipid classes. TAG, Triacylglycerol; DAG, diacylglycerol; PC, phosphatidylcholine; MAG, monoacylglycerol; SE, steryl ester. The production (mg/g_*CDW*_) of total SE **(C)** and SE species **(D)** from OE **(D)** and KI **(E)** strains, shown by the normalized abundance. All data are expressed as the mean of the triplicates originating from three independent cultures, except data from T66ASATb-KI at T2 that originate from two independent cultures. Error bars represent the standard deviation. The bars are separated into groups, each with a particular SE and time points. For each group, the bars with different lowercase letters on the top are statistically significantly different from each other (*P* < 0.05), based on one-way analysis of variance (ANOVA), followed by *post hoc* Tukey test.

The knockin strains AlASATa-OE, AlASATb-OE, T66ASATa-KI, T66ASATb-KI were cultivated using strain 30-1 as a control strain. In strain 30–1, *crtIBY* is disrupted by a *ble* expression cassette without any other genes (Rau et al., 2021). All five strains grew similarly when they were cultivated in shake flasks using glucose as the carbon source. Samples from two time points, marked T1 and T2, were collected and analyzed (Figure 5A). As expected from the previous results, neither the FA composition, nor the lipid content, showed any significant difference between the cultivated strains (Supplementary Tables 6, 7), and more than 95% of total lipid was TAG (Figure 5B). The amounts of PC, MAG, or TAG were not significantly different for the tested strains (Supplementary Table 8), while the total SE level and proportion were significantly higher in AlASATb-OE and T66ASATb-KI, compared to 30-1 (Figures 5B,C). There was also a small, but significant decrease in the proportion and amount of DAG at T2 for all knockin strains but AlASATa-OE (Figure 5B and Supplementary Table 8).

As for the KO-experiment, we then looked for the lipid species that showed statistically significant increases in either AlASATa-OE or AlASATb-OE at T1 or T2. As before, the amount of SE (16:0) and SE (22:6) were changed (Figure 5D, Table 2, and Supplementary Table 9). AlASATb-OE had accumulated a significantly higher amount of SE (16:0) and SE (22:6) than the control strain at both time points. Similarly, AlASATa-OE had accumulated more SE (16:0) and SE (22:6) at T1 and more SE (16:0) at T2, than the control strain. Moreover, AlASATb-OE had a higher amount of SE (16:0) and SE (22:6) than AlASATa-OE. These results indicate that the ASAT activity of AlASATb and, to a lesser extent, AlASATa, contribute to the accumulation of SE.

**TABLE 2:** The relative amounts (%) of lipid species in OE strains.

Time point	T1			T2		
Strain	AlASATa-OE	AlASATb-OE	AlASATb-OE	AlASATa-OE	AlASATb-OE	AlASATb-OE

Control strain	30-1	30-1	AlASATa-OE	30-1	30-1	AlASATa-OE
SE (16:0)	238 ± 25	355 ± 68	148 ± 17	211 ± 61	305 ± 81	146 ± 21
SE (22:6)	144 ± 14	236 ± 34	164 ± 16	ns	209 ± 14	175 ± 32
TG (16:0/22:6/22:6)	87 ± 0	ns	ns	ns	ns	ns
TG (22:5/22:6/22:6)	77 ± 6	ns	ns	ns	ns	ns

*Control strains are set to 100%. All values are expressed as mean ± the standard deviation of the triplicates originating from three independent cultures. ns: the amount of the lipid species was not significantly different for the two strains.*

The results for the strains expressing the T66 enzymes showed that expression of both enzymes resulted in increased amounts of each of the two SEs (Figure 5E Table 3, and Supplementary Table 9). Moreover, expression of *T66ASATb* resulted in more SE than *T66ASATa*. These results indicate that the enzymes from T66 also have ASAT activities. As for the KO strains, the impact on other lipid species were mostly insignificant (Supplementary Table 9). Two TAG species showed statistically significant reduction in AlASATa-OE at T1, but not at T2 (Table 2). DG(16:0/22:6) showed a slight increase in T66ASATb-KI at T1, while three TAG and three DAG species, showed statistically significant reduction in T66ASATa-KI or/and T66ASATb-KI at T1 or/and T2 (Table 3).

**TABLE 3:** The relative amounts (%) of lipid species in KI strains.

Time point	T1			T2		
Strain	T66ASATa-KI	T66ASATb-KI	T66ASATb-KI	T66ASATa-KI	T66ASATb-KI	T66ASATb-KI

Control strain	30-1	30-1	T66ASATa-KI	30-1	30-1	T66ASATa-KI
SE (16:0)	400 ± 61	389 ± 33	ns	306 ± 118	389 ± 117	ns
SE (22:6)	150 ± 37	190 ± 20	130 ± 20	128 ± 10	153 ± 9	121 ± 6
DG (16:0/16:0)	ns	ns	ns	65 ± 7	53 ± 10	ns
DG (16:0/22:6)	ns	122 ± 4	ns	65 ± 2	52 ± 13	ns
DG (14:0/22:6)	ns	ns	ns	50 ± 1	32 ± 11	ns
TG (20:5/22:6/22:6)	ns	87 ± 2	91 ± 4	ns	ns	ns
TG (22:6/22:6/22:6)	75 ± 8	ns	ns	ns	ns	ns
TG (22:5/22:6/22:6)	76 ± 10	80 ± 5	ns	ns	ns	ns

*Control strains are set to 100%. All values are expressed as mean ± the standard deviation of the triplicates originating from three independent cultures, except data from T66ASATb-KI at T2, originating from two independent cultures. ns: the amount of the lipid species was not significantly different for the two strains.*

### The Effect of the Expression of *AlASATa*, *AlASATb*, *T66ASATa*, or *T66ASATb* in SR21 on Squalene Accumulation

**FIGURE 6:**
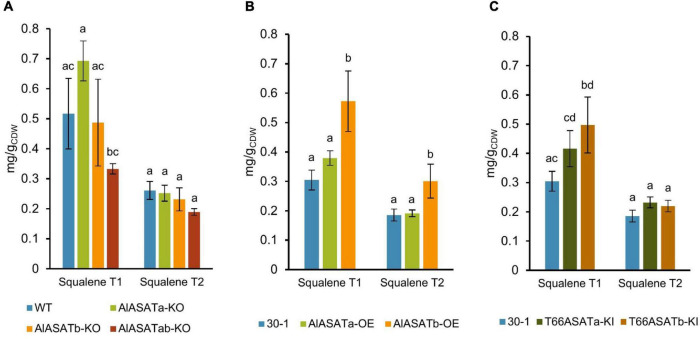
The production of squalene from the **(A)** KO, **(B)** OE, and **(C)** KI strains in milligram per gram cell dry weight (mg/g_*CDW*_). All data are expressed as the mean of the triplicates originating from three independent cultures, except data from T66ASATb-KI at T2, which originate from two independent cultures; Error bars represent the standard deviation. The bars are separated into groups, each with a particular SE and time points. For each group, the bars with all the lowercase letters different on the top are statistically significantly different from each other (*P* < 0.05), based on one-way analysis of variance (ANOVA), followed by *post hoc* Tukey test.

Squalene is a key intermediate of sterols and SE synthesis (Aasen et al., 2016). Since our results clearly indicated that *AlASATa* and *AlASATb* were involved in SE accumulation, we also analyzed cells from the same cultivations and sampling points for their squalene content to see if the expression of the two genes and their homologs from T66 would affect squalene accumulation in SR21. Of the deletion strains, only the double knockout mutant displayed significantly different squalene levels compared to the wild type strain (Figure 6A). Strain AlASATb-OE produced significantly more (up to 88% higher) squalene than strain 30-1 at both time points (Figure 6B), while overproducing AlASATa had no significant effect on the level of squalene (Figures 6B,C). On the other hand, the strains expressing the T66 enzymes both showed similar and elevated squalene levels, but they were lower than those of AlASATb-OE (Figure 6C). In addition, even though this was two separate cultivation rounds, it might be noteworthy that the control strain 30-1, with inactivated carotenoid biosynthesis, produced less squalene than the wild type strain.

## Discussion

Membrane-bound DGAT-related enzymes can be divided into five clearly separated groups (Xu et al., 2021), and from Figure 2 it is seen that the DGAT2-like protein cluster becomes very diverse when the microalgae and thraustochytrid sequences are included. The acyl-CoA:sterol acyl transferase enzymes from species like *Saccharomyces* and humans have been found to be part of the DGAT1 cluster. No putative ASAT-encoding genes have previously been suggested for either SR21 or T66. The results from the present study suggest that AlASATa, T66ASATa, AlASATb, and T66ASATb display ASAT activity and contribute to the formation of SEs. The four proteins all contain the multiple trans-membrane domains found in DGAT1-like proteins, but otherwise they belong to the DGAT2-like proteins. In *Haematococcus pluvialis*, a putative DGAT2 HpDGTT2 was found to display LPAAT activity but not DGAT activity, and this was proposed to be a consequence of evolution (Ma et al., 2020). The four ASAT proteins identified in this study might have developed in a parallel manner, suggesting that the proteins encoded by their ancestral genes might have obtained ASAT activity and lost most of their original DGAT activity through a similar evolutionary process. Alternatively, an ancestral protein with broad substrate specificity might have been duplicated and the substrate spectrum of the copies have since diverged to accept fewer substrates. Indeed, proteins with DGAT activity can have other activities and accept other substrates than DAG, such as the WS activity of the *T. aureum* bifunctional DGAT2 protein TaDGAT2 (Zhang et al., 2013), the acyl-CoA:retinol acyltransferase (ARAT), the MGAT activity of DGAT1 from mouse (Yen et al., 2005), and the PES activity of PES1 and 2 from Arabidopsis, that also harbor DGAT activity (Lippold et al., 2012). In this study, a decrease in DAG at T2 was observed when the *AlASATb* was overexpressed and when any of the T66 ASATs were produced, and there was a trend toward more DAG at T2 in AlASATb-KO (Supplementary Table 8). This might suggest that these four ASAT proteins are able to use DAG as substrate in addition to sterols.

It has been reported that acyl-CoA:acyltransferases can have a substrate preference for specific FAs. In *Nannochloropsis oceanica*, NoDGAT2A, 2C, and 2D were identified to prefer saturated FAs, polyunsaturated FAs, and monounsaturated FAs, respectively (Xin et al., 2017). In this study, we found that the relative ratios of SE (22:6) to SE (16:0) was higher than for these two FAs as part of total FA or the most abundant TAGs. However, other SE species that were not identified could also contain C16:0 or C22:6, making it difficult to use our data to discuss preference for a specific FA. Seven sterols have been identified in SR21, with cholesterol (∼62%) and lathosterol (∼31%) being the two most abundant (Yoshida et al., 2020). Although cholesterol and lathosterol have identical molecular masses, their stereochemical bond distribution might affect the efficiency for enzymes to use them as substrates. Based on their masses, SE (16:0) and SE (22:6) both can contain either cholesterol or lathosterol skeletons, or mixtures of both. A preference for sterols was shown for the *Arabidopsis thaliana*, AtSAT1 enzyme that preferred cycloartenol, followed by b-sitosterol, lanosterol, and stigmasterol (Chen et al., 2007). However, since the two SEs are composed of both the most abundant sterols and FAs, our results do not show if the enzymes have any preferences.

The branching points in the biosynthesis of sterols are shown in Figure 1, where carotenoids are included since they share the same pathway (Aasen et al., 2016). ASAT disruption in yeast has been found to increase the amount of free sterols such as ergosterol (Zweytick et al., 2000; Sorger et al., 2004) and lanosterol (Foresti et al., 2013). Moreover, additional cholesterol enhanced squalene accumulation in human cells (Gill et al., 2011). In both yeast and humans, β-hydroxy β-methylglutaryl-CoA reductase (HMGR) and squalene mono-oxygenase (SM) are the two rate-limiting steps in sterol synthesis pathway, and both can be regulated post-translationally by sterol-dependent proteasomal degradation (Song et al., 2005; Gill et al., 2011). When the sterol levels are increased in human or yeast cells, SM appears to be a bottleneck for sterol synthesis, so the squalene accumulation is enhanced and ASAT disruption increased squalene production in yeast (Zweytick et al., 2000). In this study, the expression of *AlASATb* and *T66ASATb* in SR21 presumably decreased the amount of their sterol substrates, but the squalene accumulation was enhanced. Similarly, inactivating both AlASATa and AlASATb decreased the squalene accumulation. This indicates that the regulation of this pathway in SR21 is different to the previously studied species. The pathway in Figure 1 also suggest that a decreased production of carotenoids would result in more squalene accumulation, while our results indicate that the wild type strain accumulates more squalene than strain 30-1 where *crtIBY* has been disrupted. This implies that the relationship between carotenoid and squalene synthesis involves more than competition for common precursors.

Currently, the primary commercial source of squalene is shark liver oil, and the amount that can be harvested in a sustainable manner is limited and cannot meet an increasing demand (Gohil et al., 2019). SR21 and T66 have been shown to utilize low-cost feedstock to produce DHA and squalene simultaneously, indicating that they may be an economic competitive source of sustainable squalene for the low-cost market (Patel et al., 2019, 2020). In this study, the expression of *AlASATb* or *T66ASATb* was shown to enhance squalene production. The study illustrates the potential for DGAT2-like protein to confer various functions and suggests a way to improve squalene production in thraustochytrids.

## Data Availability Statement

The original contributions presented in the study are included in the article/Supplementary Material, further inquiries can be directed to the corresponding author/s.

## Author Contributions

E-MR, ZB, KK, IA, and HE conceived and designed the experiments and performed the experiments. E-MR, ZB, KK, IA, PB, and HE interpreted the results, reviewed, and edited the manuscript. E-MR prepared the original draft. All authors read and approved the manuscript.

## Conflict of Interest

The authors declare that the research was conducted in the absence of any commercial or financial relationships that could be construed as a potential conflict of interest.

## Publisher’s Note

All claims expressed in this article are solely those of the authors and do not necessarily represent those of their affiliated organizations, or those of the publisher, the editors and the reviewers. Any product that may be evaluated in this article, or claim that may be made by its manufacturer, is not guaranteed or endorsed by the publisher.

## Footnotes

1
http://n2t.net/addgene:117226

2
http://n2t.net/addgene:35126
