# Supplementary material for: Overexpression of Two New Acyl-CoA:Diacylglycerol Acyltransferase 2-Like Acyl-CoA:Sterol Acyltransferases Enhanced Squalene Accumulation in Aurantiochytrium limacinum
Source: Front Microbiol. 2022 Jan 25;13:822254. doi: 10.3389/fmicb.2022.822254 (PMC8821962; doi:10.3389/fmicb.2022.822254)
Supplement: Supplementary file 1 [file Data_Sheet_1.pdf]

Supplementary Material

Supplementary Table S1. Strains used in this study.

**Strain**	**Description**	**Reference**
SR21	Wild-type *Aurantiochytrium limacinum* SR21 (ATCC® MYA-1381™)	ATCC®
T66	Wild-type *Aurantiochytrium* sp. T66 (ATCC® PRA-276™)	ATCC®
30-1	Transgenic strain derived from SR21 with *crtIBY* disrupted by *ble* expression cassette	Rau at al., 2021
AlASATa-KO	Transgenic strain derived from SR21 with *AlASATa* disrupted by *ble* expression cassette	This study
AlASATb-KO	Transgenic strain derived from SR21 with *AlASATb* disrupted by *ble* expression cassette	This study
AlASATab-KO	Transgenic strain derived from SR21 with *AlASATa and AlASATb* disrupted by *ntc* expression cassette and *ble* expression cassette, respectively	This study
AlASATa-OE	Transgenic strain derived from SR21 with *crtIBY* disrupted by *AlASATa* expression cassette	This study
AlASATb-OE	Transgenic strain derived from SR21 with *crtIBY* disrupted by *AlASATb* expression cassette	This study
T66ASATa-KI	Transgenic strain derived from SR21 with *crtIBY* disrupted by *T66ASATa* expression cassette	This study
T66ASATb-KI	Transgenic strain derived from SR21 with *crtIBY* disrupted by *T66ASATb* expression cassette	This study

Supplementary Table S2. Plasmids used in this study.

Plasmids	Description	Reference
pAG36	Amp^R, *nat*	Goldstein and McCusker, 1999
pEMR26	Amp^R, GAPDH promoter-*ble*-GAPDH terminator expression cassette flanked by *crtIBY* homologous sequences	Rau at al., 2021
pEMR30	Kan^R, pCR™Blunt II-TOPO® containing GAPDH promoter (rear part)-*ble* -2A-*AlASATa*-GAPDH terminator (front part)	This study
pEMR31	Kan^R, pCR™Blunt II-TOPO® containing GAPDH promoter (rear part)-*ble* -2A-*AlASATb*-GAPDH terminator (front part)	This study
pEMR32	Kan^R, pCR™Blunt II-TOPO® containing GAPDH promoter (rear part)-*ble* -2A-*T66ASATa*-GAPDH terminator (front part)	This study
pEMR33	Kan^R, pCR™Blunt II-TOPO® containing GAPDH promoter (rear part)-*ble* -2A-*T66ASATb*-GAPDH terminator (front part)	This study
pEMR34	Amp^R, GAPDH promoter-*ble*-2A-*AlASATa*-GAPDH terminator expression cassette flanked by *crtIBY* homologous sequences	This study
pEMR35	Amp^R, GAPDH promoter-*ble*-2A-*AlASATb*-GAPDH terminator expression cassette flanked by *crtIBY* homologous sequences	This study
pEMR36	Amp^R, GAPDH promoter-*ble*-2A-*T66ASATa*-GAPDH terminator expression cassette flanked by *crtIBY* homologous sequences	This study
pEMR37	Amp^R, GAPDH promoter-*ble*-2A-*T66ASATb*-GAPDH terminator expression cassette flanked by *crtIBY* homologous sequences	This study
pEMR38	Amp^R, GAPDH promoter-*ble*-GAPDH terminator expression cassette flanked by *AlASATa* homologous sequences	This study
pEMR39	Amp^R, GAPDH promoter-*ble*-GAPDH terminator expression cassette flanked by *AlASATb* homologous sequences	This study
pEMR40	Amp^R, GAPDH promoter-*nat*-GAPDH terminator expression cassette flanked by *AlASATa* homologous sequences	This study
pHE579	Amp^R, GAPDH promoter-*nat*-GAPDH-terminator expression cassette	This study
pHE583	Amp^R, GAPDH promoter-*nat*-GAPDH-terminator expression cassette flanked by *crtIBY* homologous sequences	This study
pUC19_GZG	Amp^R, GAPDH promoter-*ble*-GAPDH-terminator expression cassette	Faktorová et al., 2020

Supplementary Table S3. Primers used in this study.

Primer short name	Primer	Sequence 5'→3'	Underlined region
1	137698outF	TGTGGCCTTAATAGGAAACCTTGG	
2	137698seq1	CTGCTTAGTTGGGGCAAGCAC	
3	43553outF	GCTCGAGGTCCGCTTAATTC	
4	43553outR	GCGACTTTTCGTTGTTGATTAG	
5	21crtUFcheck	TGGCAGAGCTCATCAGTTTG	
6	SR21crtDR	ATTGTCACAGGGCGAACG	
7	21crt-midR	CGGCCGTGTTCATATAAGAG	
8	137698seq3	AGAATAAAAGCACCACACCATTC	
9	43553seq3	ACCCGAATTTTGGCGCCAAATAC	
10	2778seq2	AGCAAAGTCATGACGGTGGTTC	
11	3952seq3	GGGTCGTCGGCGTTAATGAC	
12	KI-1-BbvcI	TAGCTAGCGCTGAGGGTTG	
13	KI2957-2-2a	GTCTCCTGCTTGCTTGAGCAGAGAGAAGTTCGTGGCTCCGGATCCGTCCTGCTCCTCGGC	partial 2A sequence
14	KI137698-3	GCCACGAACTTCTCTCTGCTCAAGCAAGCAGGAGACGTGGAAGAAAACCCCGGTCCTATGGTCATTAGTTTCCTCTTTAG	partial 2A sequence
15	KI137698-4	TACATGTCGACTCTAGAGGATCCCCCTAAATGGCGTTCAAGGTC	
16	KI43553-3	GCCACGAACTTCTCTCTGCTCAAGCAAGCAGGAGACGTGGAAGAAAACCCCGGTCCTATGGCGGAGGGCGTCGAG	partial 2A sequence
17	KI43553-4	TACATGTCGACTCTAGAGGATCCCCTTAATAGTCGATGAAGTCGAGCTCGTG	
18	KI2778-3n	GCCACGAACTTCTCTCTGCTCAAGCAAGCAGGAGACGTGGAAGAAAACCCCGGTCCTATGCTGGCGCGCAAGGTGGG	partial 2A sequence
19	KI2778-4n	TACATGTCGACTCTAGAGGATCCCCCTATGCGCTAGGTAGGGTACAGTTGGTTG	
20	KI3952-3	GCCACGAACTTCTCTCTGCTCAAGCAAGCAGGAGACGTGGAAGAAAACCCCGGTCCTATGGGCGCGAGCGAGGAG	partial 2A sequence
21	KI3952-4	TACATGTCGACTCTAGAGGATCCCCCTATTTGTCGATAAAGGAGAGCGTGTGTG	

22	KI2957-5	GGGGATCCTCTAGAGTCGAC
23	KI2957-6	CAGAAATACTAGTTTTGTGAATGAAAAGAGATGATAAAAG
24	Y1-137698 f	GTAAGGAGAAAATACCGCATCAGGCGCCCTTTGCTGATGAGATTTGCGATTTG
25	Y1-137698 r	GTAGTCATATGCTCGTCTCCCTGCAGGGATTCCCGTTACGCTAAAGAGG
26	Y3-137698 f	TGTGAAATTGTTATCCGCTGCGGCCGCATTGTGCCGTGGTTTCTAGC
27	Y3-137698 r	GTATGTTGTGTGGAATTGTGAGCGGCTTAAGCGCGCCAAGTGGAATCTGTC
28	Y1-43553 fnn	GTAAGGAGAAAATACCGCATCAGGCGCCATGGCGGAGGGCGTCGAG
29	Y1-43553 rnn	GTAGTCATATGCTCGTCTCCCTGCAGGGCGAGACAACCGCAGCAAGTG
30	Y3-43553 fn	TGTGAAATTGTTATCCGCTGCGGCCGCAATTCCATACTTTATGCCTGATGAATGG
31	Y3-43553 rn	GTATGTTGTGTGGAATTGTGAGCGGCTTAAGTTAATAGTCGATGAAGTCGAGCTCGTG
32	Y4 f	CCGCTCACAATTCCACACAACATAC
33	Y4 r	AGCCGGTGAGCGTGGGTCTCGCGGTATCATTGCAGCACTGGGGCC
34	klon nat F	GCAAGAACAAGAAGCCCGGAAATGGGTACCACTCTTGAC
35	klon nat R	CTTTGATCTGACCCTAGTAGCTTAGGGGCAGGGCATGCTC
36	137698RT f	GTTCGCACAACACCTGAACC
37	137698RT r	TAGTTGCCGAAAGCGTGAAC
38	43553RT f	ATGGATGGGCGTCTGTGATG
39	43553RT r	ACGATACCGAGACCTTGCTG
40	SR21tubF2	TGTTGAGAACGCTGATGAGG
41	SR21tubR2	CGAGCTTACGGAGGTCAGAG

Supplementary Table S4. Accession numbers of the ASAT-encoding genes in this study.

Gene Name	Database	Accession	Region
T66002778.1 (*T66ASATa*)	Genbank	KQ758850.1	complement(10719593..10722430)
T66003952.1 (*T66ASATb*)	Genbank	KQ758850.1	complement(15218107..15220764)
T66011424.1	Genbank	KQ758830.1	17018..18142
T66007335.1	Genbank	KQ758891.1	complement(455514..457049)
T66010588.1	Genbank	KQ758821.1	440865..442517
T66004124.1	Genbank	KQ758850.1	15875787..15877328
T66001158.1	Genbank	KQ758850.1	4241211..4242809
Aurli1_137698 (*AlASATa*)	JGI[a]	137698	
Aurli1_43553 (*AlASATb*)	JGI	43553	
Aurli1_84317	JGI	84317	
Aurli1_115416	JGI	115416	
Aurli1_4822	JGI	4822	

[a]https://mycocosm.jgi.doe.gov/Aurli1/Aurli1.home.html

Supplementary Table S5. Proteins used for the phylogenetic tree construction.

Species and protein family	Genbank Accession Number
Acinetobacter baylyi WS/DGAT	AAO17391.1
Arabidopsis thaliana DGAT1	CAB44774.1
Arabidopsis thaliana DGAT2	NP_566952.1
Arabidopsis thaliana DGAT3	AAK06873.1
Arabidopsis thaliana PES1	NP_564662.1
Arabidopsis thaliana PES2	NP_566801.1
Arabidopsis thaliana WS/DGAT	OAO89882.1
Arachis hypogaea DGAT3	AAX62735.1
Aurantiochytrium limacinum SR21 Aurli1_115416	Aurli1_115416
Aurantiochytrium limacinum SR21 Aurli1_137698 AlASATa	Aurli1_137698
Aurantiochytrium limacinum SR21 Aurli1_43553 AlASATb	Aurli1_43553
Aurantiochytrium limacinum SR21 Aurli1_4822	Aurli1_4822
Aurantiochytrium limacinum SR21 Aurli1_84317	Aurli1_84317
Aurantiochytrium sp. T66 T66001158.1	T66001158.1
Aurantiochytrium sp. T66 T66002778.1 T66ASATa	T66002778.1
Aurantiochytrium sp. T66 T66003952.1 T66ASATb	T66003952.1
Aurantiochytrium sp. T66 T66004124.1	T66004124.1
Aurantiochytrium sp. T66 T66007335.1	T66007335.1
Aurantiochytrium sp. T66 T66010588.1	T66010588.1
Aurantiochytrium sp. T66 T66011424.1	T66011424.1
Caenorhabditis elegans DGAT1	CAB07399.2
Caenorhabditis elegans DGAT2	NP_505413.1
Chlamydomonas reinhardtii DGAT2	XP_001694904.1
Danio rerio DGAT2	NP_001025367.1

Danio rerio MGAT	NP_001008626.2
Glycine max DGAT2	ACU20344.1
Homo sapiens ASAT1	NP_003092.4
Homo sapiens ASAT2	NP_003569.1
Homo sapiens MGAT	AAK84178.1
Homo sapiens DGAT1	NP_036211.2
Homo sapiens DGAT2	AAQ88896.1
Mus musculus DGAT1	NP_034176.1
Mus musculus DGAT2	NP_080660.1
Mus musculus MGAT	AAI06136.1
Mycobacterium tuberculosis WS/DGAT	NP_217646.1
Nicotiana tabacum DGAT1	AAF19345.1
Oryza sativa DGAT1	AAW47581.1
Oryza sativa DGAT2	NP_001047917.1
Oryza sativa DGAT3	AAS98422.1
Ostreococcus tauri DGAT2	CAL58088.1
Phaeodactylum tricornutum DGAT1	ADY76581.1
Phaeodactylum tricornutum DGAT2	AFM37314.1
Phaeodactylum tricornutum WS/DGAT	XP_002184474.1
Ricinus communis DGAT2	NP_001310616.1
Saccharomyces cerevisiae ASAT1	AAT92940.1
Saccharomyces cerevisiae ASAT2	AAC49441.1
Saccharomyces cerevisiae DGAT2	NP_014888.1
Thraustochytrium aureum DGAT2	AGF92151.1
Thraustochytrium roseum WS/DGAT	ASA49417.1
Zea mays DGAT1	ABV91586.1

Supplementary Table S6. Fatty acid compositions of SR21 strains. Data are expressed as the mean ±standard deviation of the triplicates originated from three independent cultures.

g/l[a]							
Strain (time point)	C14:0	C16:0	C18:0	C20:5	C22:5	C22:6	Total
WT (T1)	0.21±0.01	2.73±0.2	0.06±0	0.01±0	0.43±0.03	1.19±0.12	4.62±0.35
AlASATa-KO (T1)	0.19±0.01	2.42±0.15	0.05±0	0.01±0	0.38±0.02	1.03±0.03	4.08±0.21
AlASATb-KO (T1)	0.19±0.01	2.23±0.13	0.05±0	0.01±0	0.35±0.01	0.99±0.04	3.83±0.18
AlASATab-KO (T1)	0.2±0.01	2.34±0.02	0.05±0	0.01±0	0.38±0	1.08±0.02	4.07±0.04
WT (T2)	0.33±0	4.74±0.26	0.11±0.01	0.02±0	0.82±0.03	2.19±0.09	8.21±0.37
AlASATa-KO (T2)	0.3±0.02	4.47±0.14	0.1±0	0.02±0	0.79±0.03	2.13±0.08	7.81±0.26
AlASATb-KO (T2)	0.3±0.01	3.97±0.06	0.09±0	0.02±0	0.7±0.02	1.88±0.05	6.97±0.09
AlASATab-KO (T2)	0.33±0.04	4.39±0.22	0.1±0.01	0.02±0	0.78±0.04	2.1±0.13	7.72±0.42

%[b]						
Strain (time point)	C14:0	C16:0	C18:0	C20:5	C22:5	C22:6
WT (T1)	4.45±0.18	59.15±0.81	1.2±0.1	0.19±0.01	9.22±0.22	25.78±0.77
AlASATa-KO (T1)	4.62±0.08	59.37±0.83	1.33±0.1	0.21±0.02	9.26±0.07	25.2±0.57
AlASATb-KO (T1)	5.03±0.14	58.35±0.87	1.36±0.07	0.23±0.01	9.22±0.26	25.82±0.75
AlASATab-KO (T1)	4.91±0.2	57.56±0.34	1.35±0.03	0.24±0	9.35±0.03	26.59±0.21
WT (T2)	4.03±0.14	57.73±0.87	1.31±0.01	0.26±0.01	9.98±0.21	26.7±0.6
AlASATa-KO (T2)	3.86±0.15	57.22±0.48	1.24±0.02	0.25±0.02	10.17±0.21	27.27±0.37
AlASATb-KO (T2)	4.35±0.13	56.99±0.67	1.32±0.02	0.29±0.01	10.02±0.1	27.03±0.58
AlASATab-KO (T2)	4.23±0.28	56.89±0.27	1.28±0.14	0.27±0.03	10.08±0.04	27.26±0.31

g/l[a]							
Strain (time point)	C14:0	C16:0	C18:0	C20:5	C22:5	C22:6	Total
30-1 (T1)	0.21±0.01	3.12±0.1	0.07±0	0.01±0	0.58±0.03	1.61±0.08	5.6±0.2
AlASATa-OE (T1)	0.22±0.04	3.28±0.22	0.07±0.01	0.01±0	0.56±0.04	1.56±0.13	5.7±0.42
AlASATb-OE (T1)	0.19±0	2.85±0.13	0.07±0	0.01±0	0.52±0.01	1.46±0.04	5.09±0.16
T66ASATa-KI (T1)	0.21±0	3.16±0.23	0.07±0	0.01±0	0.53±0.05	1.52±0.06	5.52±0.31
T66ASATb-KI (T1)	0.21±0.01	3.04±0.2	0.07±0.01	0.01±0	0.54±0.04	1.56±0.08	5.43±0.3
30-1 (T2)	0.33±0.02	5.41±0.11	0.12±0	0.03±0	1.09±0.04	2.89±0.13	9.86±0.28
AlASATa-OE (T2)	0.31±0.01	5.12±0.62	0.11±0.01	0.02±0	0.92±0.11	2.56±0.28	9.06±1.02
AlASATb-OE (T2)	0.27±0	4.61±0.16	0.11±0	0.02±0	0.88±0.04	2.41±0.1	8.3±0.29
T66ASATa-KI (T2)	0.29±0.01	4.54±0.28	0.1±0	0.02±0	0.86±0.05	2.35±0.13	8.17±0.47
T66ASATb-KI (T2)	0.3±0.02	5.39±0.33	0.11±0	0.02±0	0.98±0.1	2.74±0.21	9.53±0.65

%[b]						
Strain (time point)	C14:0	C16:0	C18:0	C20:5	C22:5	C22:6
30-1 (T1)	3.73±0.12	55.66±0.49	1.2±0.04	0.22±0.02	10.39±0.11	28.8±0.63
AlASATa-OE (T1)	3.79±0.28	57.58±0.27	1.29±0.14	0.23±0.03	9.76±0.04	27.35±0.31
AlASATb-OE (T1)	3.65±0.2	55.95±0.77	1.33±0.08	0.24±0.01	10.23±0.25	28.6±0.35
T66ASATa-KI (T1)	3.88±0.27	57.33±1.39	1.3±0.04	0.23±0.02	9.62±0.37	27.63±1.05
T66ASATb-KI (T1)	3.9±0.43	56.05±0.82	1.27±0.12	0.22±0.01	9.9±0.34	28.65±0.24
30-1 (T2)	3.33±0.15	54.83±0.57	1.24±0.05	0.27±0.01	11.04±0.06	29.29±0.48
AlASATa-OE (T2)	3.46±0.29	56.53±0.43	1.25±0.07	0.26±0.03	10.2±0.15	28.29±0.1
AlASATb-OE (T2)	3.25±0.16	55.51±0.26	1.29±0.08	0.29±0.01	10.62±0.15	29.05±0.18
T66ASATa-KI (T2)	3.53±0.14	55.61±0.5	1.24±0.04	0.28±0.02	10.55±0.04	28.78±0.35
T66ASATb-KI (T2)	3.1±0.03	56.57±0.42	1.15±0.12	0.24±0.02	10.25±0.55	28.7±0.39

[a]Grams per liter of the culture. [b]Percentage of total fatty acids. The FAs less than 0.01 g/l were considered as background signals and were not shown

Supplementary Table S7. Lipid content (lipid/dried cell, g/g) of SR21 strains. Data are expressed as the mean ±standard deviation of the triplicates originated from three independent cultures unless specified.

Strain \ Time point	T1	T2
WT	0.67±0.02	0.87±0.01
AlASATa-KO	0.64±0.04	0.87±0.01
AlASATb-KO	0.7±0.01	0.82±0.04
AlASATab-KO	0.69±0.01	0.83±0.01
30-1	0.74±0.04	0.77±0.03
AlASATa-OE	0.69±0.04	0.79±0
AlASATb-OE	0.72±0.01	0.78±0.03
T66ASATa-KI	0.72±0.06	0.81±0.05
T66ASATb-KI	0.71±0.01	0.77±0.02[a]

[a]Mean ±variation of the duplicate originated from two independent cultures.

Supplementary Table S8. Content of lipid classes in SR21 strains. Data are expressed in milligram per gram cell dry weight (mg/g CDW), distribution (%) and as the mean ±standard deviation of the triplicates originated from three independent cultures.

mg/g$_{CDW}$				
Strain (time point)	TAG	PC	DAG	MAG
WT (T1)	589.13±32.45	15.24±1.64	3.16±0.17	0.36±0.02
AlASATa-KO (T1)	667.5±34.81	10.08±2.43	2.52±0.47	0.46±0.1
AlASATb-KO (T1)	545.94±11.03	15.15±1.51	3.19±0.17	0.53±0.16
AlASATab-KO (T1)	683.09±34.3	9.16±2.27	2.12±0.2	0.37±0.03
WT (T2)	570.55±48.82	17±1.54	3.23±0.89	0.46±0.18
AlASATa-KO (T2)	644.12±30.43	9.53±2.52	2.94±0.65	0.54±0.16
AlASATb-KO (T2)	575.53±10.28	16.54±1.07	2.79±0.89	0.47±0.22
AlASATab-KO (T2)	628.9±29.37	9.64±3.16	3.23±1.53	0.48±0.14

%				
Strain (time point)	TAG	PC	DAG	MAG
WT (T1)	96.87±0.21	2.5±0.22	0.52±0.01	0.06±0.01
AlASATa-KO (T1)	96.6±0.31	2.68±0.29	0.56±0.04	0.09±0.03
AlASATb-KO (T1)	96.51±0.19	2.88±0.14	0.54±0.1	0.08±0.02
AlASATab-KO (T1)	96.67±0.05	2.78±0.22	0.47±0.14	0.08±0.04
WT (T2)	97.99±0.44	1.48±0.37	0.37±0.06	0.07±0.01
AlASATa-KO (T2)	98.24±0.32	1.32±0.32	0.31±0.04	0.05±0.01
AlASATb-KO (T2)	98.03±0.33	1.44±0.32	0.45±0.09	0.08±0.02
AlASATab-KO (T2)	97.94±0.59	1.49±0.42	0.5±0.21	0.07±0.02

mg/g$_{CDW}$

	TAG	PC	DAG	MAG
BLE (T1)	592.42±27.39	14.66±1.24	2.05±0.11	0.37±0.04
AlASATa-OE (T1)	594.21±25.65	8.43±0.69	4.39±0.58	0.46±0.18
AlASATb-OE (T1)	562.19±17.28	15.13±0.26	2.53±0.71	0.42±0.1
T66ASATa-KI (T1)	586.39±25.74	9.37±1.68	4.4±0.68	0.44±0.16
T66ASATb-KI (T1)	564.75±31.42	16.55±1.31	2.25±0.48	0.44±0.16
BLE (T2)	609.23±29.43	9.95±0.35	3.04±0.68	0.35±0.01
AlASATa-OE (T2)	556.44±24.37	13.31±2.03	2.54±0.56	0.45±0.16
AlASATb-OE (T2)	629.83±34.96	10.13±0.88	3.01±0.47	0.45±0.13
T66ASATa-KI (T2)	568.41±20.42	13.03±0.94	2.37±0.15	0.35±0.01
T66ASATb-KI (T2)	559.95±98.36	9.28±1.5	2.36±0.35	0.4±0.08

		%		
	TAG	PC	DAG	MAG
BLE (T1)	97.14±0.21	2.4±0.19	0.34±0.01	0.06±0.01
AlASATa-OE (T1)	96.8±0.18	2.61±0.11	0.44±0.13	0.07±0.02
AlASATb-OE (T1)	96.48±0.1	2.83±0.2	0.38±0.08	0.08±0.03
T66ASATa-KI (T1)	97.02±0.17	2.31±0.25	0.44±0.1	0.08±0.03
T66ASATb-KI (T1)	97.08±0.13	2.23±0.15	0.4±0.02	0.06±0
BLE (T2)	97.74±0.18	1.39±0.06	0.72±0.1	0.08±0.03
AlASATa-OE (T2)	97.52±0.09	1.55±0.21	0.74±0.14	0.07±0.03
AlASATb-OE (T2)	97.58±0.15	1.59±0.03	0.49±0.12	0.06±0
T66ASATa-KI (T2)	97.76±0.18	1.57±0.1	0.47±0.08	0.07±0.02
T66ASATb-KI (T2)	97.6±0.65	1.68±0.58	0.43±0.14	0.07±0.03

Supplementary Table S9. Content of selected lipid species. Only compounds with statistically significant difference between WT and at least one KO mutants, 30-1 and at least one OE mutants, or 30-1 and at least one KI mutants at T1 or T2 are displayed. Data are expressed in milligram per gram cell dry weight (mg/g CDW) and as the mean ±standard deviation of the triplicates originated from three independent cultures unless specified. Normalized using response of the ISTD of known concentration.

	T1				T2			
	WT	AlASATa-KO	AlASATb-KO	AlASATab-KO	WT	AlASATa-KO	AlASATb-KO	AlASATab-KO
SE(16:0)	0.12±0.02	0.11±0.02	0±0	0±0	0.27±0.01	0.26±0.02	0.01±0.01	0±0
SE(22:6)	1.05±0.03	1.2±0.18	0.17±0.02	0.05±0.01	1.66±0.18	1.48±0.2	0.49±0.04	0.11±0.03

	T1			T2		
	30-1	AlASATa-OE	AlASATb-OE	30-1	AlASATa-OE	AlASATb-OE
SE(16:0)	0.11±0.02	0.26±0.01	0.38±0.03	0.18±0.05	0.36±0.04	0.52±0.03
SE(22:6)	1.1±0.16	1.57±0.14	2.57±0.07	1.44±0.13	1.74±0.27	2.99±0.09
TG(16:0/22:6/22:6)	1.02±0.02	0.89±0.02	1±0.08	1.14±0.09	1.03±0.06	1.16±0.08
TG(22:5/22:6/22:6)	1.34±0.05	1.02±0.04	1.19±0.09	1.41±0.09	1.14±0.09	1.33±0.17

	T1			T2		
	30-1	T66ASATa-KI	T66ASATb-KI	30-1	T66ASATa-KI	T66ASATb-KIa
SE(16:0)	0.11±0.11	0.43±0.01	0.42±0.02	0.18±0.05	0.51±0.05	0.6±0.01
SE(22:6)	1.1±0.16	1.61±0.16	2.07±0.11	1.44±0.13	1.83±0.07	2.17±0.16
DG(16:0/16:0)	0.05±0	0.06±0.01	0.06±0	0.1±0.01	0.06±0.01	0.05±0.01
DG(16:0/22:6)	1.34±0.05	1.47±0.14	1.63±0.1	2.91±0.18	1.9±0.18	1.52±0.27
DG(14:0/22:6)	0.05±0	0.06±0.02	0.05±0.01	0.08±0.01	0.04±0	0.03±0.01
TG(20:5/22:6/22:6)	0.1±0	0.1±0	0.09±0	0.12±0	0.14±0.02	0.11±0.01
TG(22:6/22:6/22:6)	2.95±0.11	2.22±0.27	2.54±0.05	2.94±0.11	3.07±0.26	2.71±0.12
TG(22:5/22:6/22:6)	1.34±0.05	1.02±0.15	1.06±0.07	1.41±0.09	1.25±0.16	1.16±0.03

[a]Mean±variation of the duplicate originated from two independent cultures.

Supplementary Figure S1

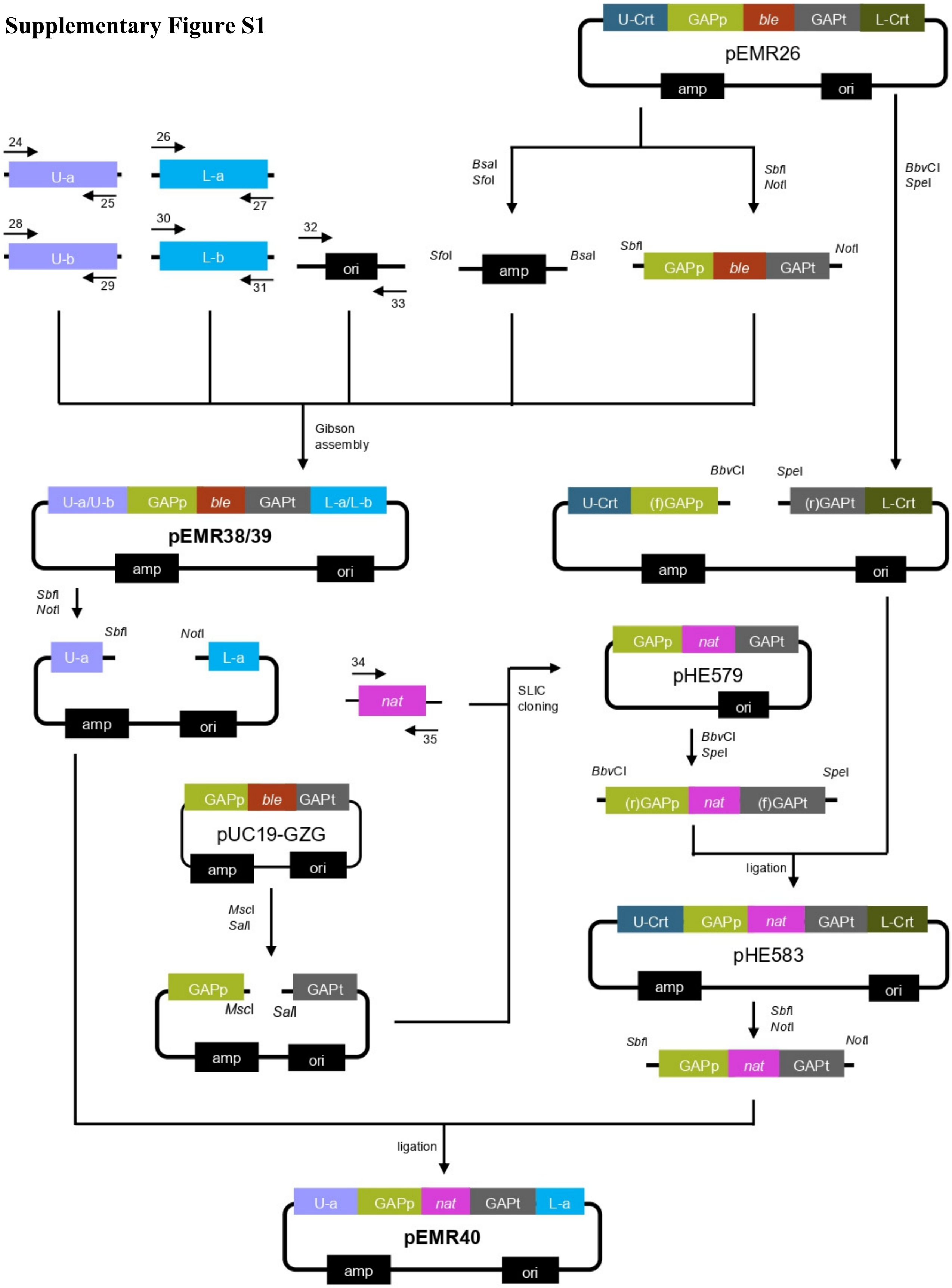

Supplementary Figure S1. Scheme of constructing pEMR38, 39 and 40. U-a and L-a: *AlASATa* homologous regions; U-b and L-b: *AlASATb* homologous regions; U-Crt/L-Crt: *crtIBY* homologous regions; GAPp: GAPDH promoter; GAPt: GAPDH terminator; (f)/(r)GAPt: the front/rear of GAPt ; (f)/(r)GAPp: the front/rear of GAPp; *ble*: Zeocin resistance gene; *nat*: nourseothricin resistance gene; amp: ampicillin resistance cassette; ori: pMB1 origin of replication; 24~33: PCR primers. U-a, L-a, U-b and L-b were amplified from SR21 genomic DNA. The fragment containing ori was amplified from pEMR26. The fragment containing *nat* was amplified from pAG36.

Supplementary Figure S2

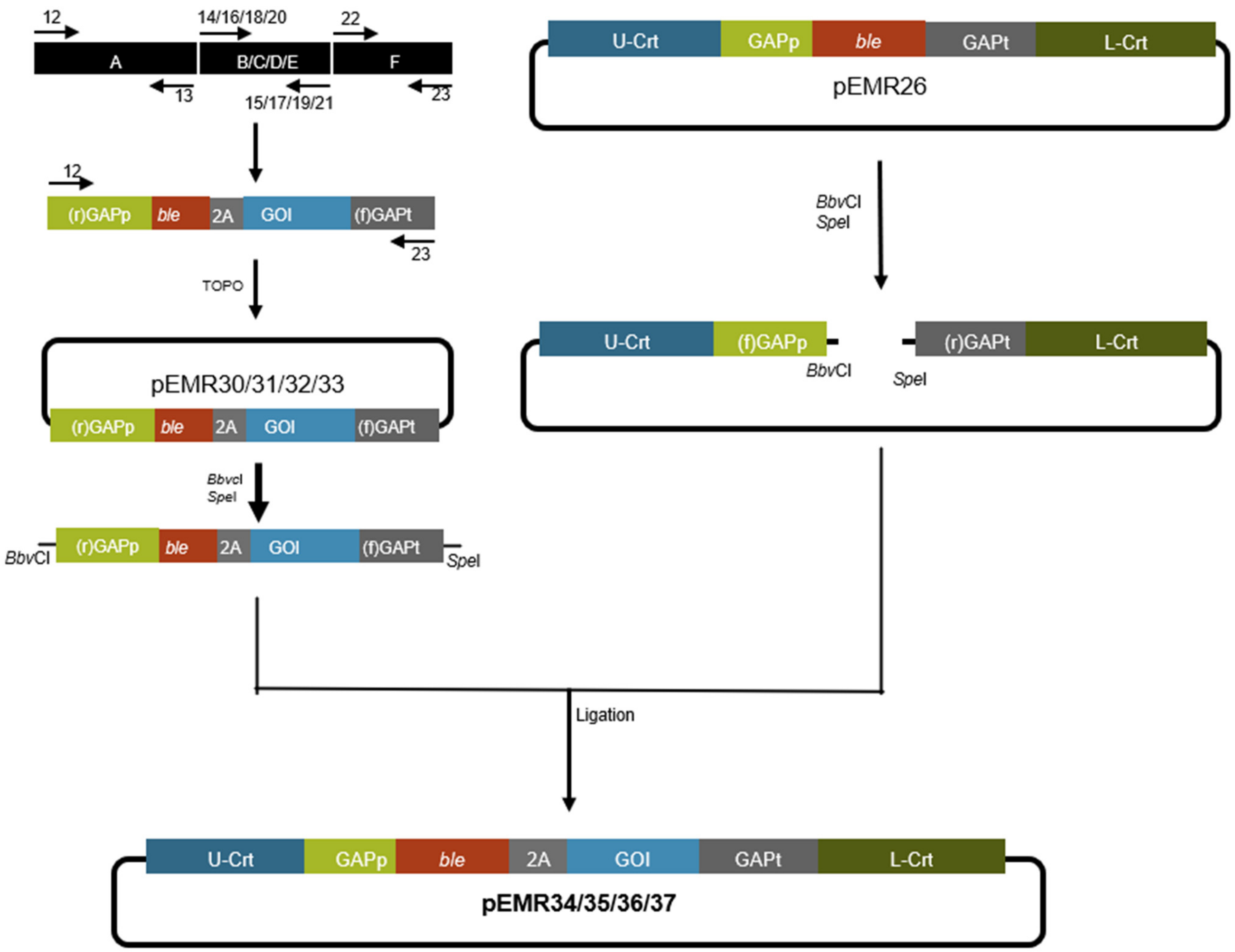

Supplementary Figure S2. Scheme of constructing pEMR34, 35, 36 and 37. U-Crt/L-Crt: *crtIBY* homologous regions; GOI: *AlASATa*, *AlASATb*, *T66ASATa* or *T66ASATb*; GAPp: GAPDH promoter; GAPt: GAPDH terminator; (f)/(r)GAPt: the front/rear of GAPt ; (f)/(r)GAPp: the front/rear of GAPp ; *ble*: Zeocin resistance gene; 2A: peptide self-cleavage sequence; 12~23: PCR primers. The PCR products A and F were amplified from pUC19-18GZG, B, C were amplified from SR21 genomic DNA and, D and E were amplified from T66 genomic DNA. Gel-Purified A, F and each of the fragments among B, C, D and E were mixed as templates for the following PCR reaction.

Supplementary Figure S3

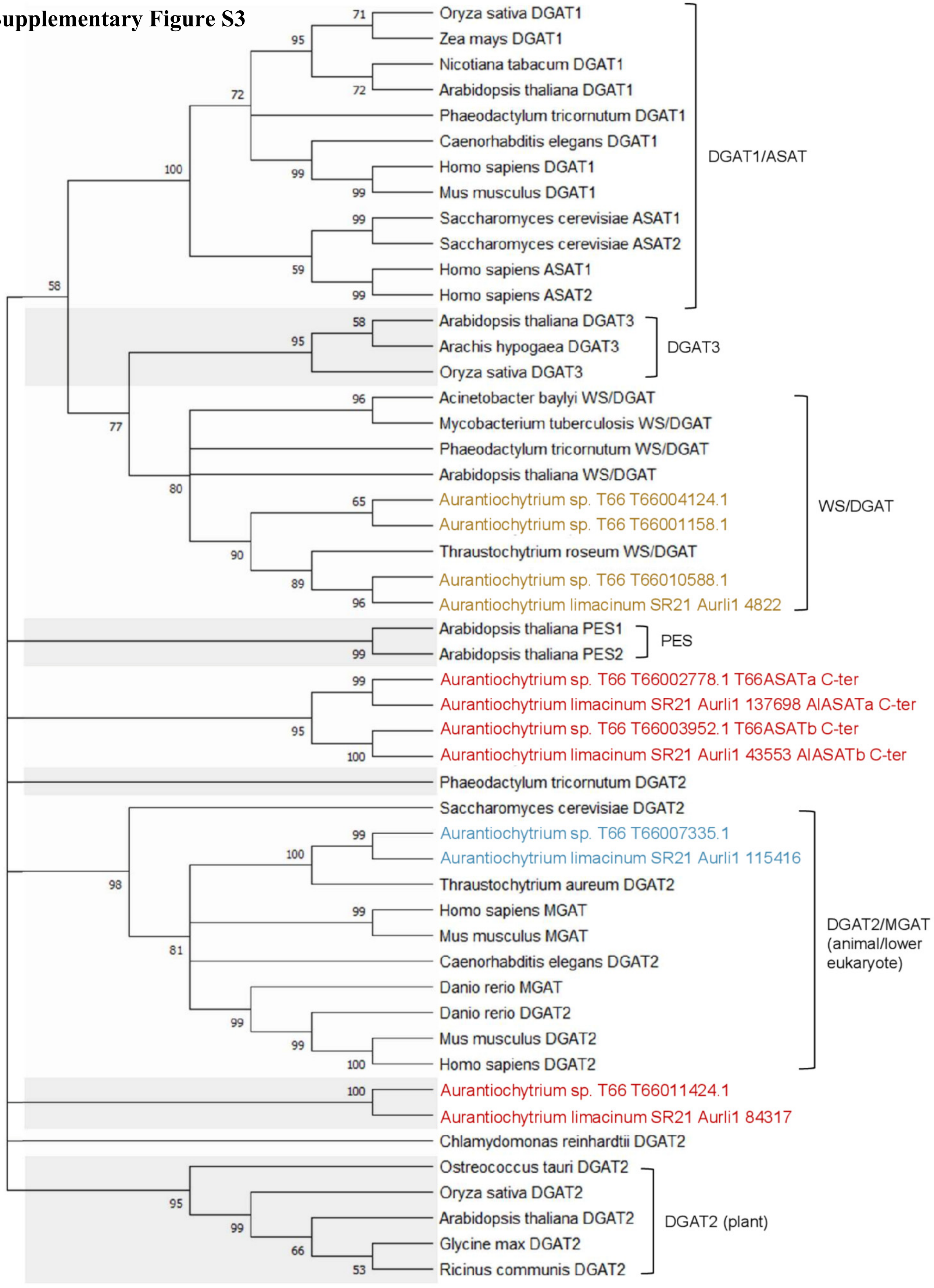

Supplementary Figure S3. Phylogenetic analysis of DGAT2s (blue), WS/DGATs (yellow) and DGAT2-like proteins (red) in T66 and SR21, and representative functionally characterized acyl-CoA:acyltransferases from different organisms. Only the C-terminal part of the four ASAT proteins were included in the analysis. The proteins' accession numbers are summarized in Supplementary Table S5. There were a total of 1114 positions in the final dataset. The percentage of replicate trees in which the associated taxa clustered together in the bootstrap test (1000 replicates) are shown next to the branches, which were collapsed if the corresponding partitions reproduced in less than 50% bootstrap replicates.

Supplementary Figure S4

a

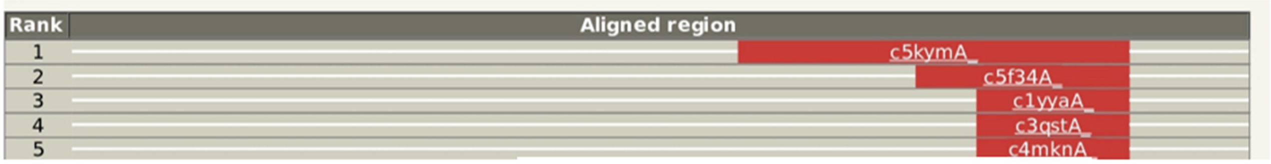

b

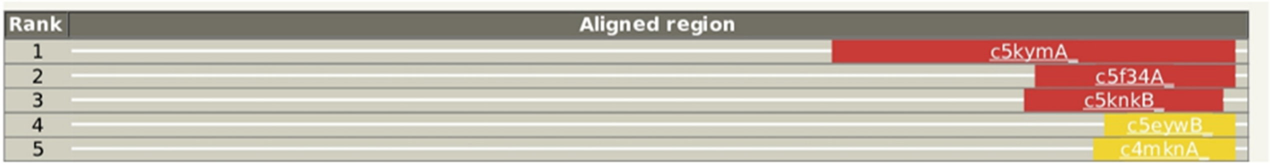

Supplementary Figure S4. Alignment of AlASATa (a); and AlASATb (b) using Phyre2 (Kelley et al., 2015). Only the C-terminal part showed significant homology to proteins with known structure.

Supplementary Figure S5

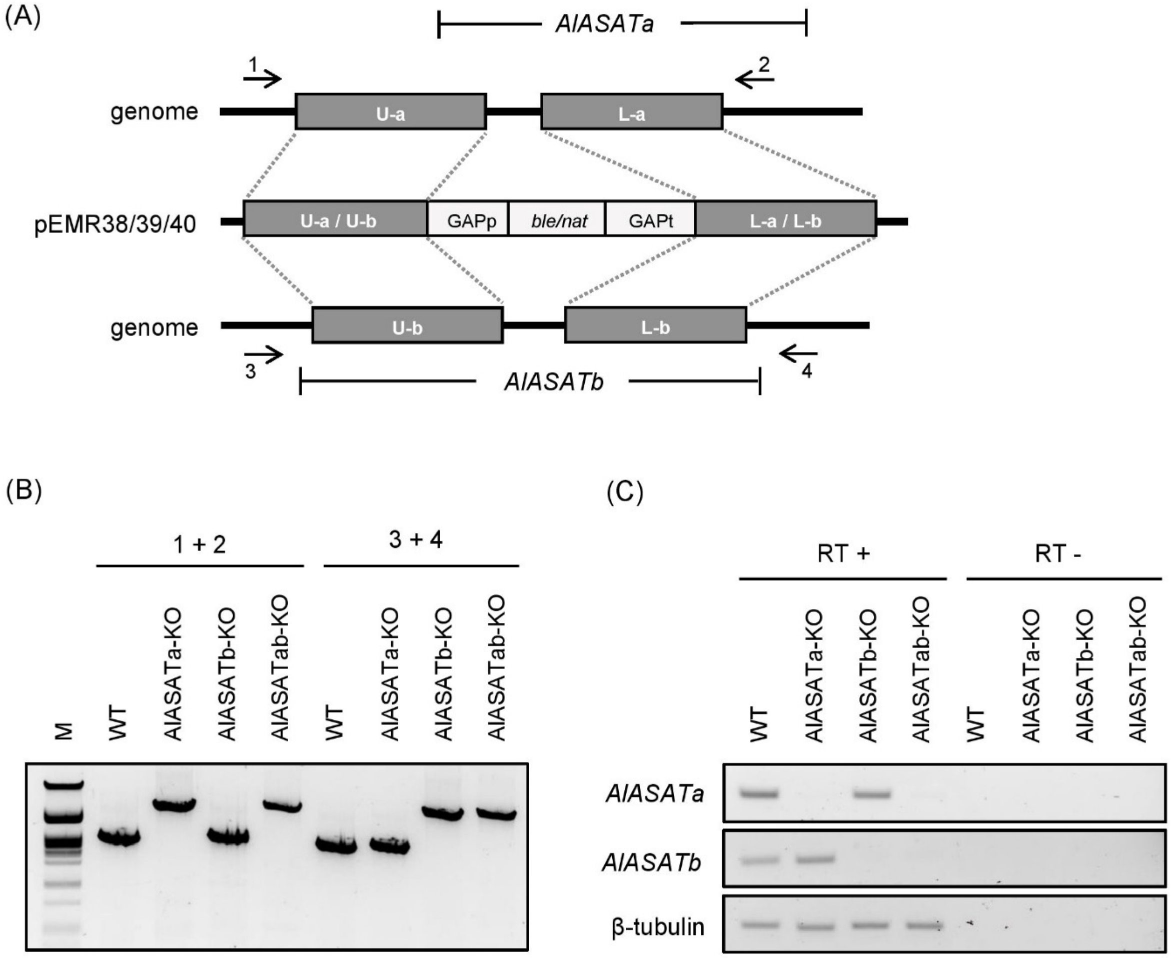

Supplementary Figure S5. (A) Scheme for how linearized pEMR38 (containing U-a, L-a and *ble*), pEMR39 (containing U-b, L-b and *ble*) or pEMR40 (containing U-a, L-a and *nat*) integrate into the SR21 genome; GAPp/t: GAPDH promoter/terminator; *ble*: Zeocin resistance gene; *nat*: nourseothricin resistance gene; Arrows: primer annealing sites. (B) Genomic PCR of indicated strains were performed with the indicated primer pairs, shown by 0.8% agarose gel electrophoresis; M: lambda-*Pst*I ladder. Refer to supplementary Figure S6 for expected lengths. (C) To verify absence of wild type genes, putative transcripts of the indicated strains were detected by RT-PCR using specific primers for partial *AlASATa*, *AlASATa* and β-tubulin gene and displayed by 2% agarose gel electrophoresis; RT-/+: template RNA before/after reverse transcription.

Supplementary Figure S6

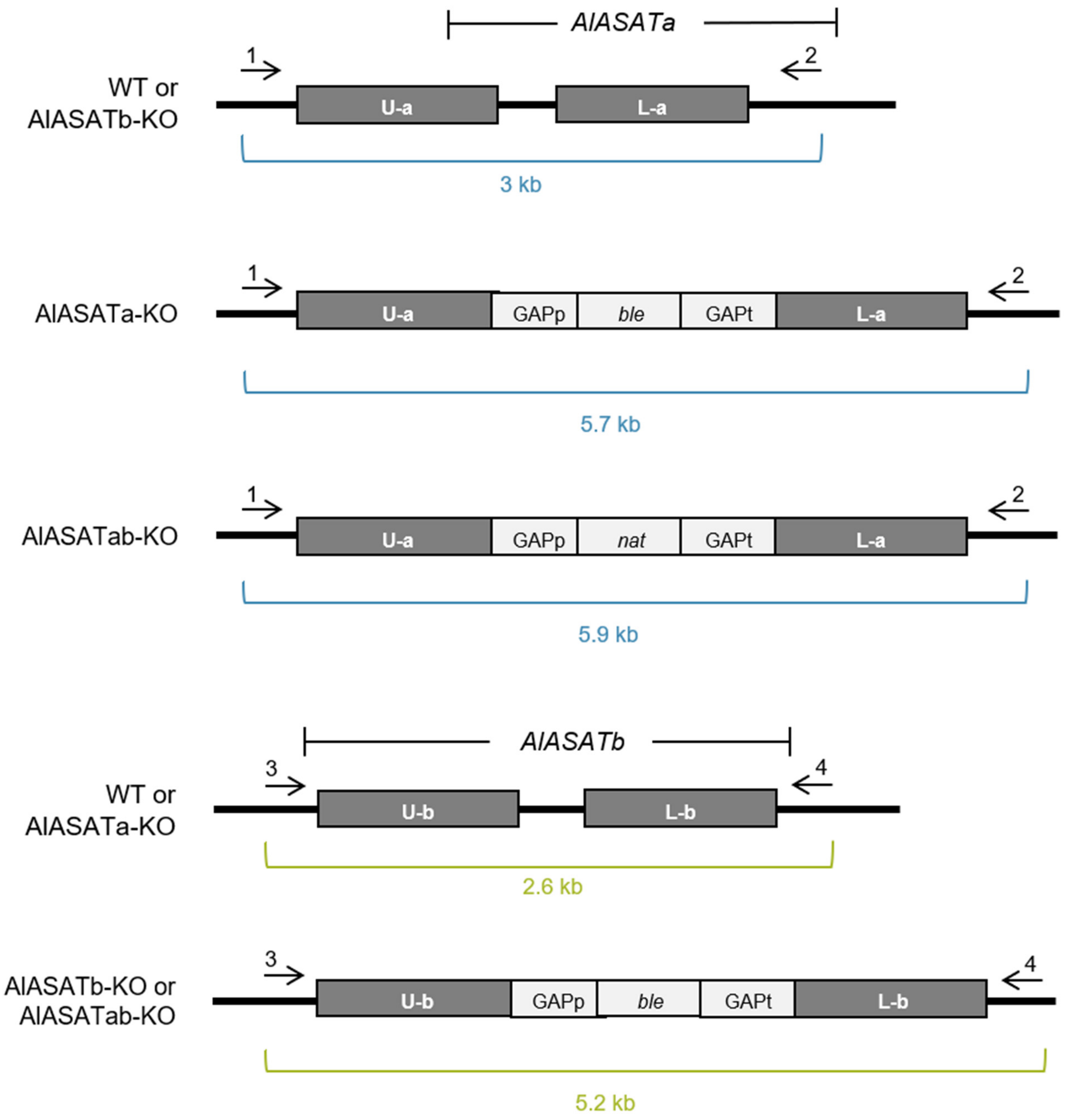

Supplementary Figure S6. Illustration of the sizes of PCR products amplified from the gnomic DNA of the indicated SR21 strains. U-a and L-a: *AlASATa* homologous regions; U-b and L-b: *AlASATb* homologous regions; GAPp/t: GAPDH promoter/terminator; *ble*: Zeocin resistance gene; *nat*: nourseothricin resistance gene; Arrows: primer annealing sites.

Supplementary Figure S7

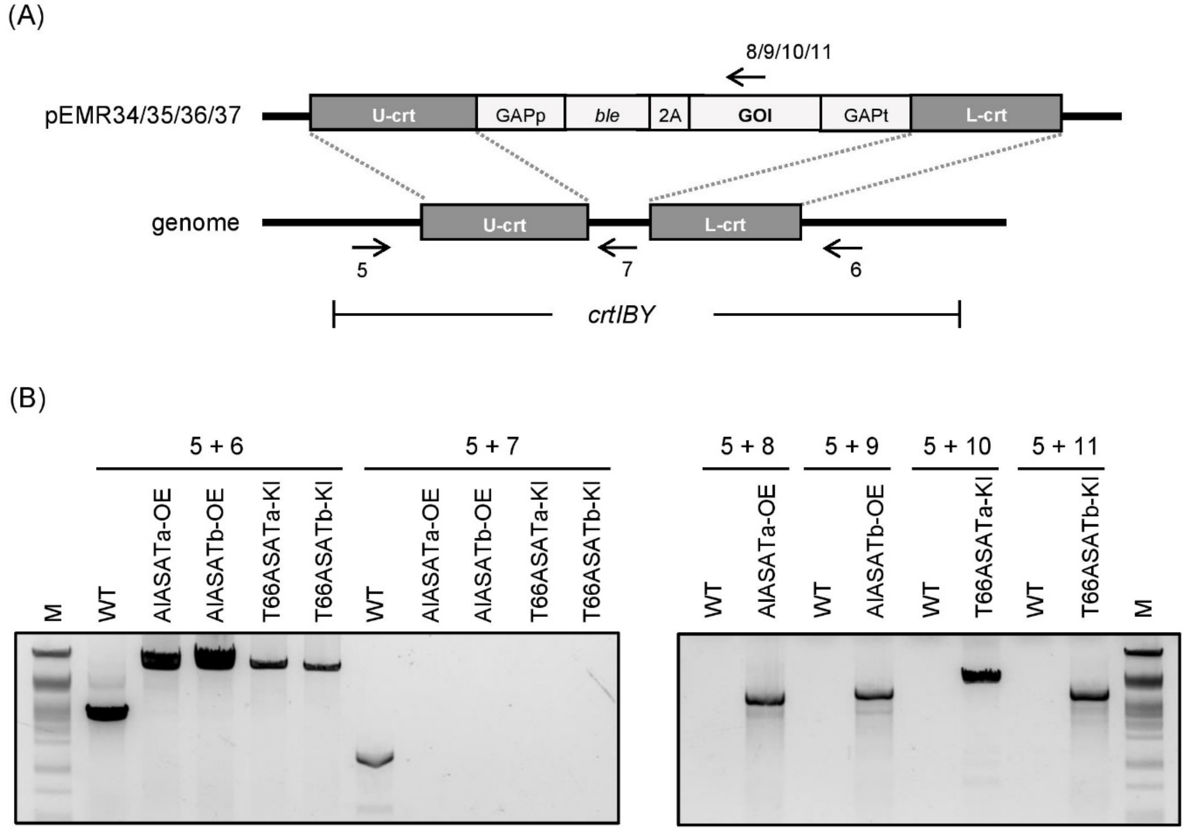

Supplementary Figure S7. (A) Scheme for linearized pEMR34, 35 36 or 37 with the gene of interest (GOI) *AlASATa*, *AlASATb*, *T66ASATa* or *T66ASATb*, respectively, integrating into SR21 genome. U-crt and L-crt: homologous regions; GAPp/t: GAPDH promoter/terminator; *ble*: zeocin resistance gene; 2A: peptide self-cleavage sequence; Arrows: primer annealing sites. (B) Genomic PCRs of indicated strains were performed with the indicated primer pairs, and separated by 0.8% agarose gel electrophoresis; M: lambda-*Pst*I ladder.

Supplementary Figure S8

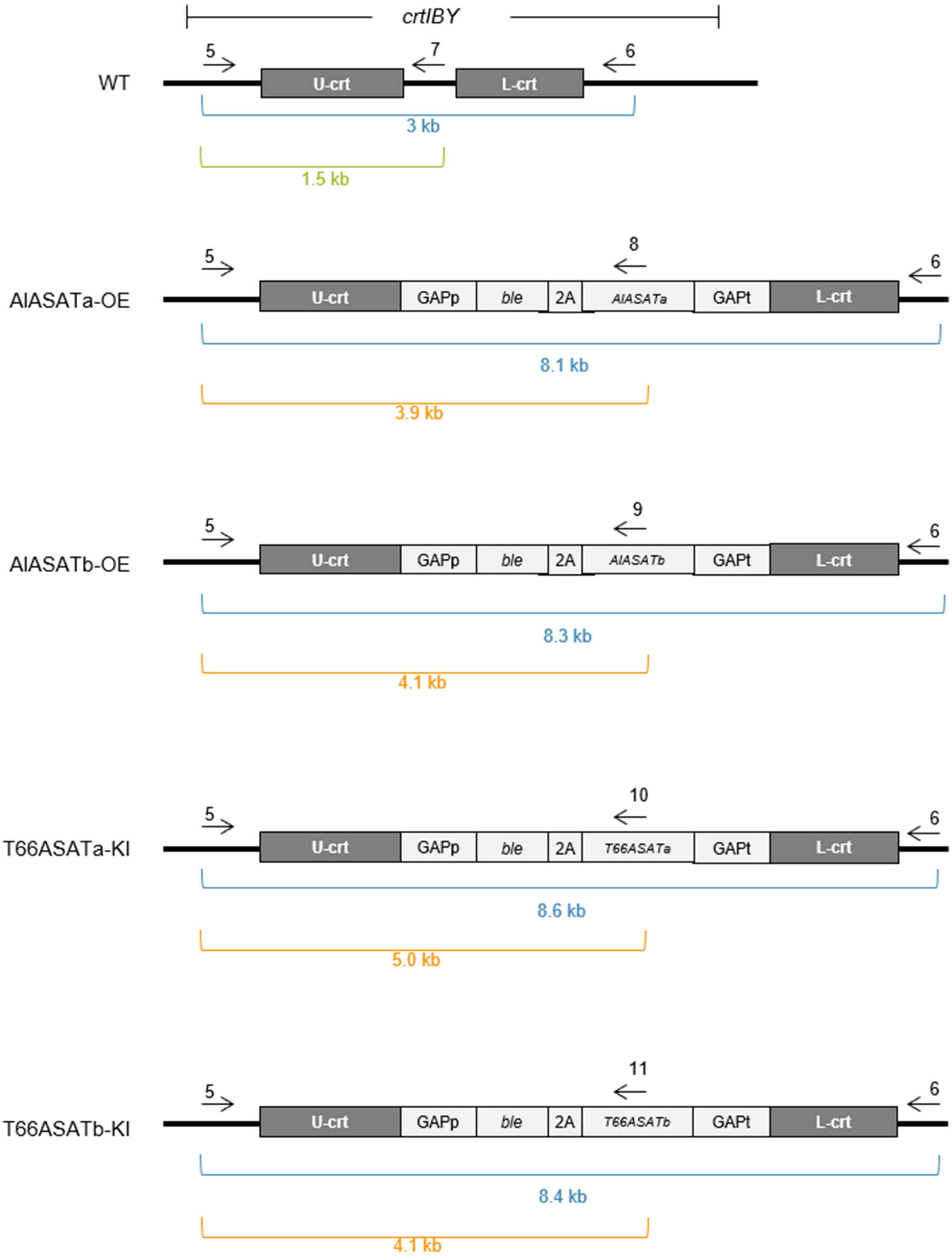

Supplementary Figure S8. Illustration of the sizes of PCR products amplified from the gnomic DNA of the indicated SR21 strains. U-Crt/L-Crt: *crtIBY* homologous regions; GAPp/t: GAPDH promoter/terminator; *ble*: Zeocin resistance gene; *nat*: nourseothricin resistance gene; 2A: peptide self-cleavage sequence; Arrows: primer annealing sites.
